# NPR1 Promotes Lipid Droplet Lipolysis to Enhance Mitochondrial Oxidative Phosphorylation and Fuel Gastric Cancer Metastasis

**DOI:** 10.1002/advs.202503233

**Published:** 2025-06-20

**Authors:** Huafeng Fu, Jie Zhang, Hengxing Chen, Haobin Hou, Huanjie Chen, Rongman Xie, Yanlei Chen, Jian Zhang, Dehua Liu, Leping Yan, Rui L. Reis, Joaquim M. Oliveira, Yulong He, Li Zhong, Qinbo Cai, Dongjie Yang

**Affiliations:** ^1^ Department of Gastrointestinal Surgery Digestive Medicine Center The Seventh Affiliated Hospital Sun Yat‐sen University Shenzhen 518107 P. R. China; ^2^ General Surgery Laboratory The First Affiliated Hospital Sun Yat‐sen University Guangzhou Guangdong 510080 P. R. China; ^3^ Guangdong Provincial Key Laboratory of Digestive Cancer Research Shenzhen 518107 P. R. China; ^4^ Center for Gastrointestinal Surgery The First Affiliated Hospital Sun Yat‐sen University Guangzhou 510080 P. R. China; ^5^ Scientific Research Center The Seventh Affiliated Hospital of Sun Yat‐sen University Shenzhen 518107 P. R. China; ^6^ 3B's Research Group I3Bs – Research Institute on Biomaterials Biodegradables and Biomimetics Headquarters of the European Institute of Excellence on Tissue Engineering and Regenerative Medicine University of Minho AvePark, Zona Industrial da Gandra Barco 4805‐017 Guimarães Portugal; ^7^ ICVS/3B's – PT Government Associate Laboratory Braga 4805‐017 Guimarães Portugal; ^8^ Research Center for Diagnosis and Treatment of Gastric Cancer Sun Yat‐sen University Guangzhou P. R. China

**Keywords:** engineered cell membrane‐derived exosome mimetics, gastric cancer, lipid droplet, lipolysis, lymph node metastasis, mitochondrial oxidative phosphorylation, natriuretic peptide receptor 1

## Abstract

Metabolic reprogramming driven by oncogenes plays a critical role in promoting and sustaining multiple steps of gastric cancer metastasis. However, the key metabolic driver of metastasis that can lead to the development of targeted therapies for preventing and treating metastatic gastric cancer remains elusive. Here, it is identified that the transmembrane guanylate cyclase, natriuretic peptide receptor 1 (NPR1), promoted gastric cancer lymph node metastasis by activating lipid droplet lipolysis and enhancing mitochondrial oxidative phosphorylation (OXPHOS). Clinical analysis reveals that elevated NPR1 protein level is correlated with increased lymph node metastasis and shorter patient survival. Functionally, NPR1 induced lipolysis of stored lipid droplets, releasing bioavailable fatty acids that are imported into mitochondria to upregulate OXPHOS, thus fueling the energy required for the metastasis of gastric cancer cells. Mechanistically, NPR1 activates protein kinase cGMP‐dependent 1 (PRKG1 or PKG), which directly bound to and activated hormone‐sensitive lipase (HSL) by phosphorylation at residues Ser^855^ and Ser^951^, thereby increasing lipolysis. Furthermore, targeted delivery of NPR1 siRNA using engineered exosome mimetics effectively suppressed gastric cancer metastasis. Taken together, these findings elucidate the NPR1‐driven metabolic mechanism underlying gastric cancer metastasis and suggest NPR1 as a promising therapeutic target for patients with metastatic gastric cancer.

## Graphical abstract

NPR1 induces lipolysis of stored lipid droplet and enhances mitochondrial oxidative phosphorylation to fuel gastric cancer metastasis. Specifically, NPR1‐mediated activation of PKG directly phosphorylates and activates HSL at Ser855 and Ser951, thereby driving lipolytic processes. Targeting NPR1 with siNPR1‐loaded engineered exosome mimetics offers a potential therapeutic strategy to inhibit gastric cancer metastasis.

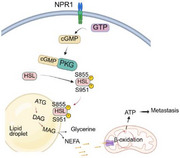

## 1 Introduction

Gastric cancer (GC) remains a significant global health challenge, ranking fifth in cancer incidence and fourth in cancer‐related mortality, with particularly high incidence rates in East Asia, Eastern Europe, and South America.^[^ ^1^ ^]^ Lymph node metastasis (LNM) is prevalent in gastric cancer, which is one of the most critical negative prognostic factors in gastric cancer. Even in patients with early gastric cancer, the prevalence of lymph node metastasis is high, ranging from 22.1% to 27.3%.^[^ ^2^ ^]^ Cancer metastasis significantly hinders the prognosis of GC patients.^[^ ^3^, ^4^ ^]^ However, the mechanisms driving LNM in GC remain poorly understood, underscoring the urgent need to elucidate the molecular mechanisms of LNM and develop therapeutic strategies targeting LNM in GC.

Metabolic dynamic changes in metastatic tumor cells represent a promising target for therapeutic intervention. Accumulating evidence supports that metabolic reprogramming is crucial for initiating and sustaining cancer metastatic cascades.^[^ ^5^, ^6^ ^]^ Primary tumor cells rely on aerobic glycolysis (Warburg effect) for energy production.^[^ ^6^ ^]^ However, extensive studies highlight the pivotal role of active mitochondrial oxidative phosphorylation (OXPHOS) in tumor metastasis. In vivo studies revealed that metastatic tumor cells possess elevated levels of OXPHOS compared to primary tumor cells.^[^ ^7^, ^8^ ^]^ Caroline et al. found that mitochondrial OXPHOS and ATP production are suppressed in primary tumors but elevated in metastatic breast cancer cells.^[^ ^9^ ^]^ Moreover, epidemiological studies showed that metformin (a mitochondrial complex I inhibitor) reduces the recurrence and metastasis of ovarian and breast cancers.^[^ ^10^, ^11^, ^12^ ^]^ Therefore, understanding the molecular mechanisms driving elevated mitochondrial OXPHOS in metastatic tumor cells may offer opportunities to prevent tumor metastasis and enhance outcomes for patients with advanced‐stage cancer.

Fatty acids (FAs) are important substrates for mitochondrial OXPHOS. Extensive evidence suggests that fatty acid oxidation (FAO) may be the preferred energy source for cancer metastatic progression. Upregulated FAO or lipid metabolism‐related genes confer a metastatic advantage for cancer cells.^[^ ^5^, ^13^, ^14^, ^15^, ^16^, ^17^ ^]^ Lipid droplets (LDs) are dynamic organelles that store neutral lipids, such as triglycerides and sterol esters, and respond to the cell's energy and lipid metabolism requirements.^[^ ^18^, ^19^ ^]^ Enzyme‐catalyzed lipolysis releases bioavailable FAs from LDs, which are imported into mitochondria for β‐oxidation and ATP production, fueling cancer metastasis.^[^ ^20^, ^21^, ^22^, ^23^ ^]^ Therefore, dysregulation of lipolysis or key enzymes involved in this process may be a mechanism promoting tumor metastasis.

Natriuretic peptide receptor 1 (NPR1) is a transmembrane guanylate cyclase that catalyzes the production of the second messenger cyclic guanosine monophosphate (cGMP) upon activation. cGMP subsequently activates downstream effectors, including protein kinase G (PKG), phosphodiesterases, and ion channels. NPR1 is an important regulator of cardiovascular homeostasis and lipid metabolism.^[^ ^24^, ^25^ ^]^ Our previous work indicates that NPR1 methylation is associated with gastric cancer progression.^[^ ^26^ ^]^ NPR1 has recently been implicated in tumor progression and drug resistance.^[^ ^27^, ^28^ ^]^ For example, NPR1 knockdown triggers cytoprotective autophagy and ROS accumulation,^[^ ^29^ ^]^ promotes angiogenesis through HIF‐1α interaction,^[^ ^30^ ^]^ and enhances stemness and drug resistance by recruiting mesenchymal stem cells^[^ ^28^ ^]^ in GC. Blocking NPR1 with antibodies has been shown to inhibit GC growth.^[^ ^31^ ^]^ However, its specific role in cancer metabolism and metastasis remains unclear.

In this study, using patient samples and mouse models, we identified NPR1 as a critical regulator of GC LNM. We demonstrated that NPR1 promotes lipolysis of stored LD to release bioavailable FAs, which are imported into mitochondria to upregulate OXPHOS and ATP production. This process is driven by PKG, a primary downstream protein kinase of NPR1, which phosphorylates HSL to drive lipolysis and ultimately fuel gastric cancer metastasis. Notably, we generated engineered cell membrane‐derived exosome mimetics for the targeted delivery of NPR1 siRNA into cancer cells, which effectively suppressed GC metastasis.

## 2 Results

### 2.1 NPR1 is Associated With Tumor Metastasis in Gastric Cancer

**Figure 1:**
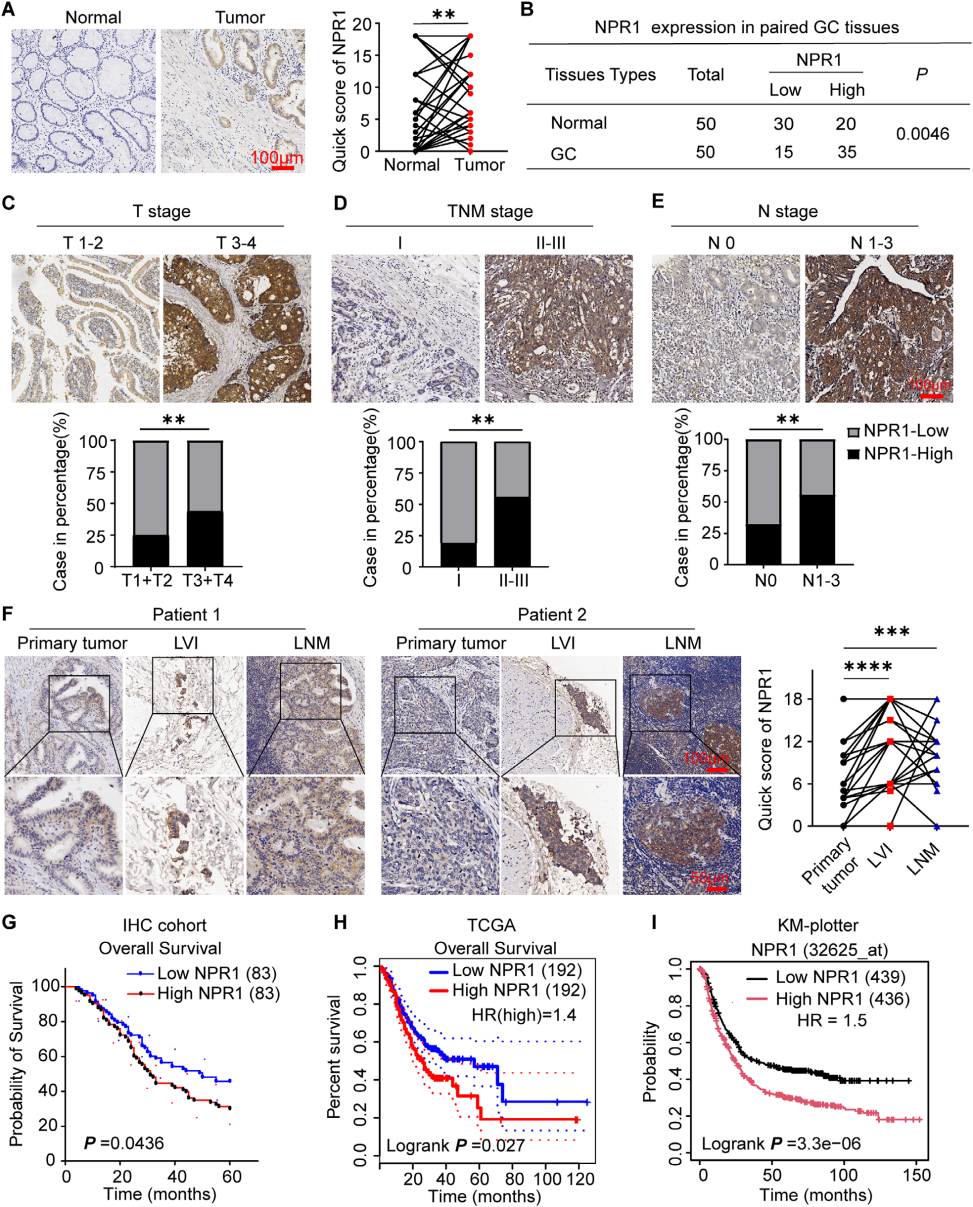
NPR1 is a tumor metastasis‐related gene in gastric cancer. A) Representative images of IHC staining for NPR1 expression in gastric cancer and adjacent normal tissue. The score was determined by assessing the strength and extent of immunopositivity, *n* = 50, *p*‐values are calculated using Wilcoxon matched‐pairs signed rank test. B) Statistical analysis of NPR1 protein expression in gastric cancer and adjacent normal tissue, *n* = 50, *p*‐values are calculated using Chi‐Squared test. C–E) Representative images of IHC staining for NPR1 and the percentages of cases with different NPR1 expression levels in T stage, lymph node metastasis, and TNM stages are shown, n (NPR1‐High) = 83, n (NPR1‐Low) = 83, *p*‐values are calculated using Chi‐Squared test. F. Representative images of IHC staining for NPR1 expression in metastatic gastric cancer and primary tumors, *n* = 30, *p*‐values are calculated using Friedman's ANOVA tests. G–I) Kaplan–Meier survival analysis of the overall survival of gastric cancer patients with low versus high NPR1 expression. Survival analysis was carried out using univariate Cox and log‐rank tests. ^*^ *p* < 0.05, ^**^ *p* < 0.01, ^***^ *p* < 0.001, ^****^ *p* < 0.0001. GC: gastric cancer. LVI: lymphovascular invasion. LNM: lymph node metastasis.

Previous studies have shown that NPR1 plays an important role in GC progression and chemoresistance; however, the role of NPR1 in GC metastasis remains unclear.^[^ ^28^, ^29^, ^30^, ^31^, ^32^ ^]^ Here, we detected the expression and distribution of NPR1 in GC tissue samples. NPR1 expression was significantly upregulated in GC compared to matched adjacent normal tissue (**Figure** **1**A,B). To determine the clinical relevance of NPR1 protein in GC patients, we explored its relationship with various clinicopathological characteristics (Table S4, Supporting Information). Patients with higher T grades (T grade 3/4) or advanced gastric cancer (TNM stage II/III) showed higher NPR1 protein levels than those with lower T grades (T grade 1/2) or early‐stage GC (TNM stage I) (Figure 1C,D). In addition, patients with lymph node metastasis exhibited higher NPR1 protein levels (Figure 1E). Analysis of paired primary tumor and lymph node metastasis tissues revealed that NPR1 protein levels were higher in lymphovascular invasion (LVI) tissues and lymph node metastasis tissues than in matched primary GC tissues (Figure 1F).

Cancer metastasis often threatens the survival of patients. In our IHC cohort, Kaplan–Meier survival analysis revealed that high NPR1 expression was significantly associated with unfavorable overall survival (OS) (log‐rank test, *p* = 0.0436) (Figure 1G). Consistently, analyses of the TCGA data set (log‐rank test, *p* = 0.0027) (Figure 1H) and the Kaplan–Meier Plotter dataset (log‐rank test, *p* < 0.001) (Figure 1I) confirmed similar results. Collectively, these results suggest that NPR1 is closely associated with GC metastasis and significantly correlates with unfavorable patient prognosis.

### 2.2 NPR1 Promotes Migration, Invasion and Lymph Node Metastasis of GC Cells

**Figure 2:**
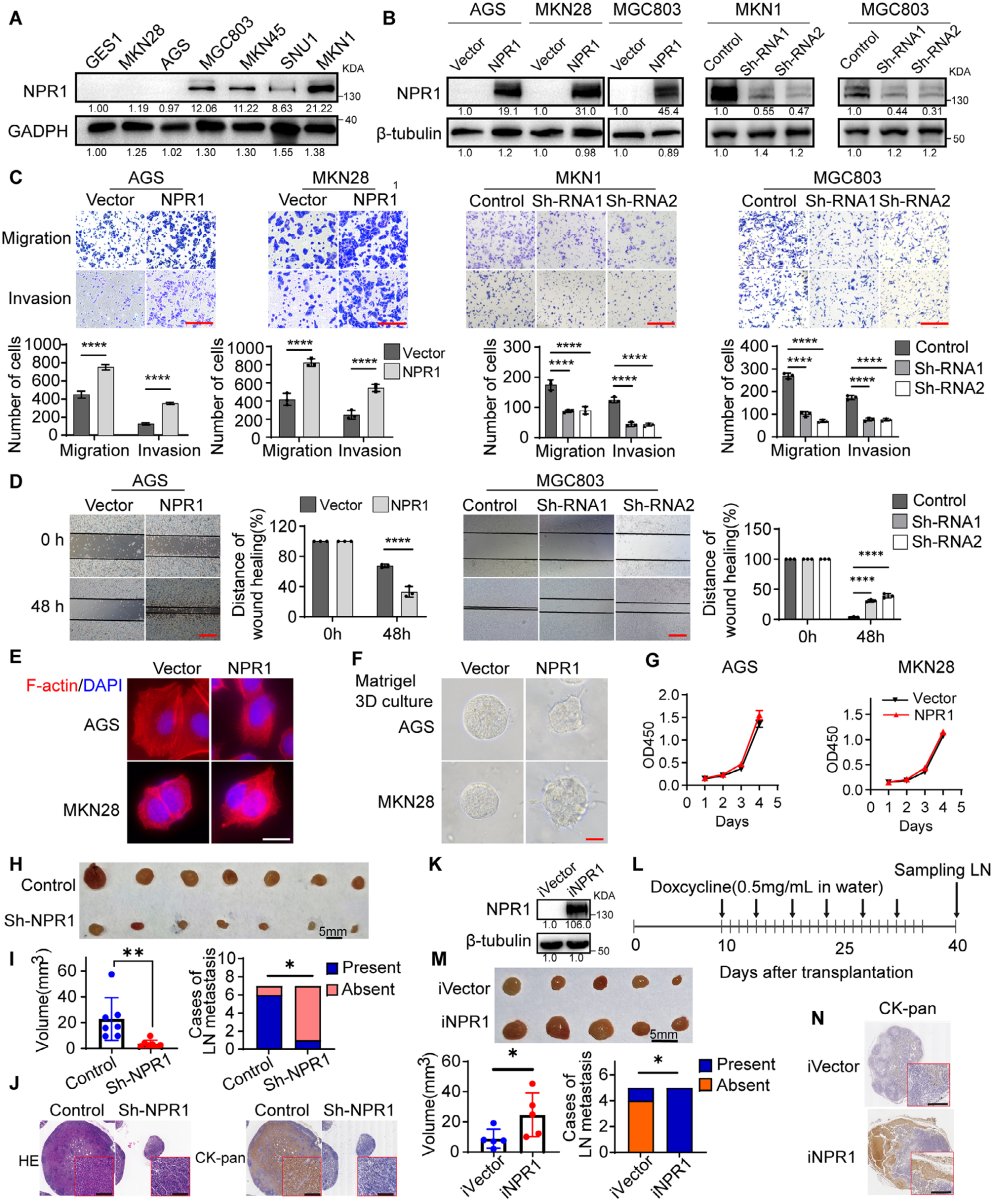
Elevated NPR1 promotes gastric cancer cell invasion and metastasis. A) Protein levels of GES1, AGS, MKN28, MGC803, MKN45, SNU1 and MKN1. B) AGS, MKN28 and MGC803 cells were transfected with NPR1 cDNA, whereas MGC803 and MKN1 cells were transfected with NPR1 shRNAs. Transfection efficiencies were assessed using western blotting. C) The migration and invasive abilities conveyed by NPR1 knockdown or NPR1 overexpression cells were measured using Transwell assay and Matrigel Invasion assay. Scale bar = 50 µm. Data presented as mean ± SD, *n* = 3, *p*‐values are calculated using two‐way ANOVA tests. D) The migration abilities conveyed by NPR1 knockdown or NPR1 overexpression cells were measured using Wound‐healing assay. Scale bar = 200 µm. Data presented as mean ± SD, *n* = 3, *p*‐values are calculated using two‐way ANOVA tests. E) Staining of F‐actin in Vector and NPR1 overexpressing cells. Scale bar = 10 µm. F) when cultured in 3D Matrigel, NPR1 overexpression cells appeared to show an aggressive morphology with protrusion of longer podosomes. Scale bar = 100 µm. G) The effect of NPR1 overexpression on the proliferation of gastric cancer cells was detected by CCK‐8 assay. Data presented as mean ± SD, *n* = 5, *p*‐values are calculated using two‐way ANOVA tests. H) Images of popliteal lymph nodes. I) Statistical analysis of the volume and the presence of metastasis in popliteal lymph nodes, *n* = 7, Mann–Whitney test was used for comparing lymph node volumes, and Fisher's Exact test was used for comparing the presence of metastasis in lymph nodes. J) The presence of metastasis in the lymph node was confirmed by H&E and CK‐pan staining. Scale bar = 100 µm. K) GC006‐03 cell was transfected with NPR1 cDNA and selected for clones that stably express NPR1 upon induction. Transfection efficiencies were assessed using western blotting. L) Schematic view of the popliteal lymph node metastasis mouse model treated by doxycycline. M) Images of popliteal lymph nodes, and statistical analysis of the volume and the presence of metastasis in lymph nodes in popliteal lymph nodes, *n* = 5, Mann–Whitney test was used for comparing lymph node volumes, and Fisher's Exact test was used for comparing the presence of metastasis in lymph nodes. N) The presence of metastasis in the lymph node was confirmed by CK‐pan staining. Scale bar = 100 µm. ^*^ *p* < 0.05, ^**^ *p* < 0.01, ^***^ *p* < 0.001, ^****^ *p* < 0.0001.

To investigate the biological function of NPR1 in GC, we stably transfected NPR1 cDNA vector to overexpress NPR1 in AGS and MKN28 cells, which showed low endogenous NPR1 levels. Conversely, two independent short hairpin RNAs (shRNAs) were employed to stably knock down NPR1 expression in MGC803 and MKN1 cells, which exhibited high endogenous NPR1 levels (**Figure** **2**A,B; Figure S1A, Supporting Information). Transwell and wound healing assays showed that NPR1 overexpression significantly enhanced the migration and invasion abilities of AGS and MKN28 cells (Figure 2C,D). In contrast, NPR1 knockdown significantly inhibited the migration and invasion abilities of MKN1 and MGC803 cells (Figure 2C,D). Immunofluorescence staining revealed that NPR1‐overexpressing cells presented more F‐actin polymerization and invadopodia formation than the control cells (Figure 2E). In 3D Matrigel culture, NPR1‐overexpressing cells appeared to show an aggressive morphology, characterized by the protrusion of longer podosomes (Figure 2F). In addition, there were no significant changes in the protein levels of E‐cadherin (an epithelial marker) or Vimentin (a mesenchymal marker) in GC cells following transient overexpression of NPR1 (Figure S1B, Supporting Information), suggesting that NPR1 does not directly induce EMT to enhance the metastatic potential of gastric cancer cells. Also, NPR1 overexpression did not significantly alter GC cell proliferation in vitro (Figure 2G).

We established a mouse popliteal lymph node metastasis model to confirm the in vivo effects of NPR1 on GC metastasis. Mice injected with NPR1‐silenced cells exhibited significantly smaller popliteal lymph nodes (LNs) compared to control mice (Figure 2H,I). H&E and CK‐pan staining were used to confirm Metastatic LNs (Figure 2J), which indicated that loss of NPR1 significantly inhibited the presence of LNM (Figure 2H,I). We cloned NPR1 cDNA into a doxycycline‐inducible lentiviral vector (pINDUCER system) and generated stable GC006‐03 GC cell expressing NPR1 upon induction (Figure 2K). The stable cell lines were injected into the foot pads of nude mice, and doxycycline (0.5 mg mL^−1^) was added to the drinking water to induce NPR1 expression 2 weeks later (Figure 2L). Results showed that the volumes of popliteal LNs were significantly larger in the NPR1 elevated group mice than in the corresponding control group mice (Figure 2M). CK‐pan staining indicated that elevated NPR1 expression significantly promoted the presence of LNM (Figure 2N). Furthermore, we also observed the metastasis‐promoting function of NPR1 in a hematogenous lung metastasis model (Figure S1C–E, Supporting Information), which indicates the metastasis‐promoting effect of NPR1 is universal. These results indicate that NPR1 enhances GC cell migration, invasion, and lymph node metastasis. Notably, the pro‐metastatic effect of NPR1 is independent of its effect on tumor proliferation.

### 2.3 NPR1 Facilitates Mitochondrial Metabolism and ATP Production

**Figure 3:**
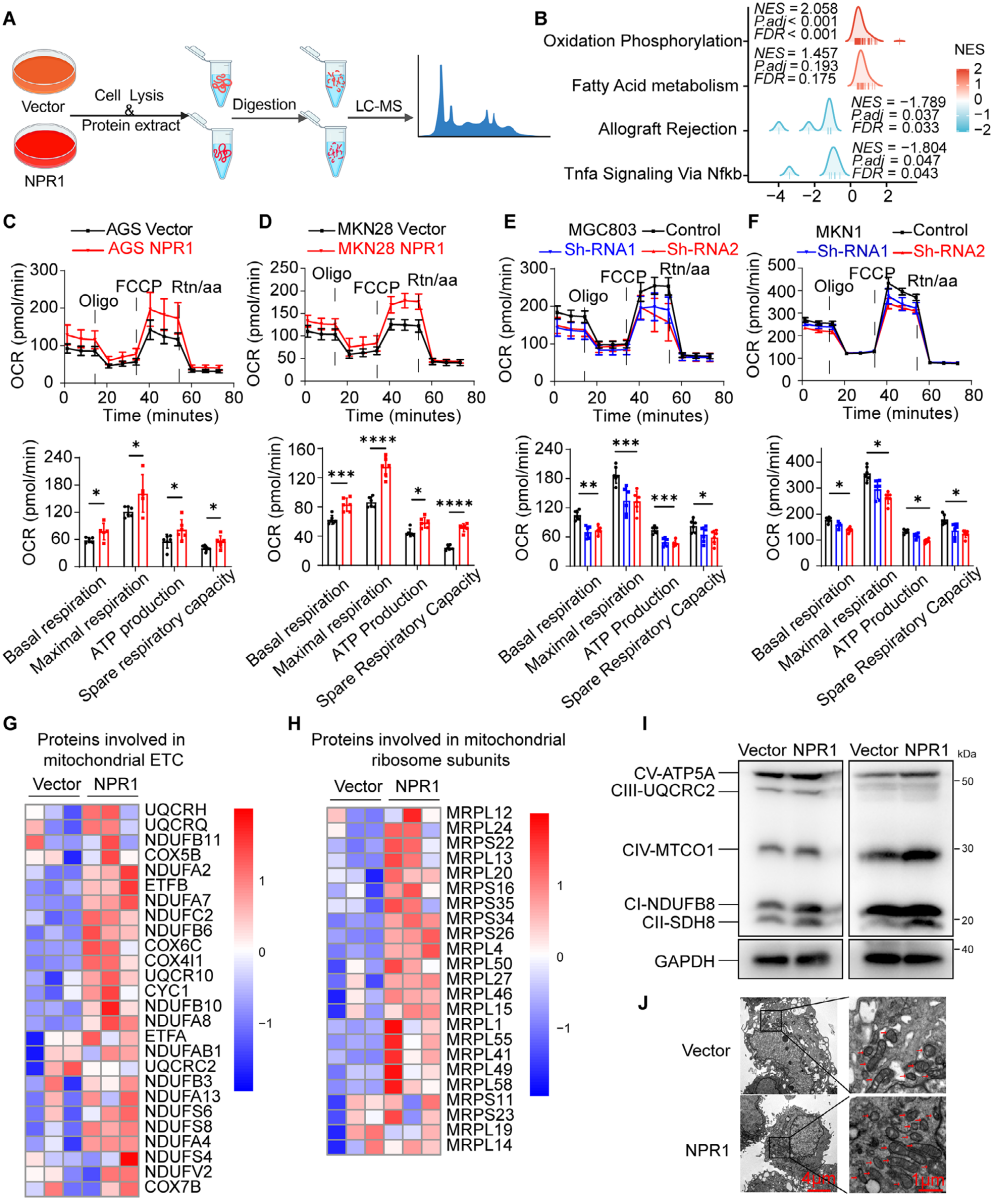
NPR1 facilitates mitochondrial metabolism and ATP production. A) Schematic view of the label‐free proteomic sequencing of NPR1 overexpression and control cells. B) GSEA analysis of the proteomic sequencing data. C–F) OCR analysis of NPR1 overexpression or knockdown cells, and statistical analysis of the mitochondrial respiration capacity and ATP production. Data presented as mean ± SD, *n* = 6, *p*‐values are calculated using two‐way ANOVA tests. G) Heat map of altered expression of proteins involved in mitochondrial electron transfer chain in cells after NPR1 overexpression. H) Heat map of altered expression of proteins involved in mitochondrial ribosome subunits in cells after NPR1 overexpression. I) The increase of mitochondrial mass by NPR1 overexpression was verified by Western blotting. J) The numbers of mitochondria in NPR1 overexpression cells were detected by transmission electron microscopy analyses. ^*^ *p* < 0.05, ^**^ *p* < 0.01, ^***^ *p* < 0.001, ^****^ *p* < 0.0001.

To explore how NPR1 regulates GC metastasis, we performed label‐free proteomic analysis on NPR1‐overexpressing and control cells (**Figure** **3**A). A total of 3243 proteins were identified (Figure S2, Supporting Information), and GSEA analysis showed that OXPHOS and fatty acid metabolism pathways ranked as the top two positively enriched pathways (Figure 3B). These findings suggest that NPR1 may be involved in mitochondrial metabolism and lipid metabolic remodeling. To verify the effects of NPR1 on mitochondrial metabolism, we measured the oxygen consumption rate (OCR) in cultured cells. NPR1 overexpression significantly increased mitochondrial respiration capacity and ATP production (Figure 3C,D), while NPR1 knockdown markedly diminished mitochondrial respiration capacity and ATP production (Figure 3E,F). Consistent with these mitochondrial respiratory phenotypes, the proteomic analysis identified upregulation of proteins involved in mitochondrial electron transfer chain (ETC) and ribosome subunits in NPR1‐overexpressing cells (Figure 3G,H). Western blot analysis verified the increase in mitochondrial mass in NPR1‐overexpressing cells (Figure 3I). Transmission electron microscopy (TEM) analysis further demonstrated increased numbers of mitochondria in NPR1‐overexpressing cells compared to control cells (Figure 3J). These results indicate that NPR1 enhances mitochondrial metabolism and ATP production in GC cells.

### 2.4 NPR1 Promotes Lipid Droplet Lipolysis to Enhance β‐Oxidation and Fuel Gastric Cancer Metastasis

**Figure 4:**
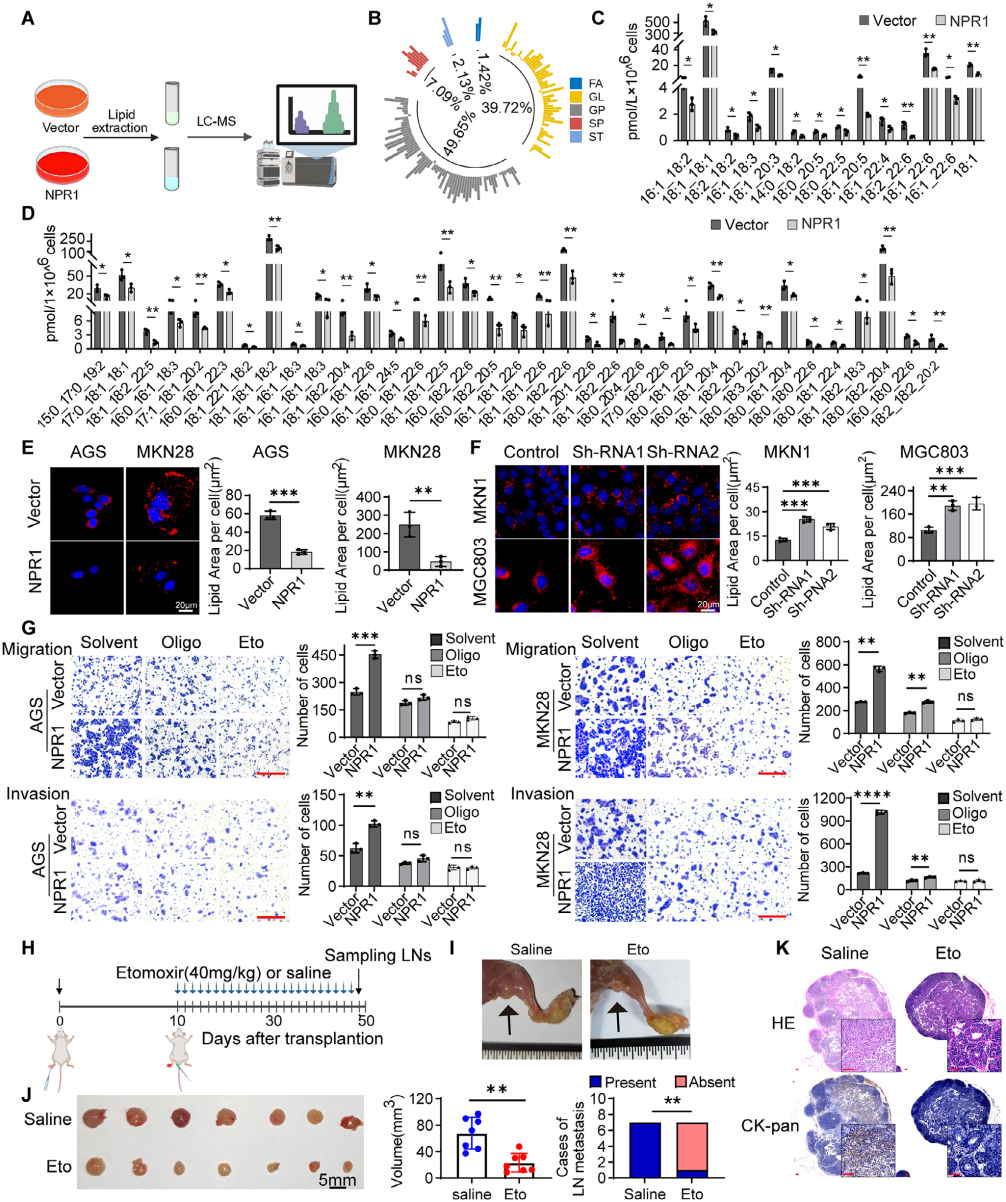
NPR1 promotes lipid droplet lipolysis to enhance β‐oxidation and fuel gastric cancer metastasis. A) Schematic view of the targeted lipidomic sequencing of NPR1 overexpression and control cells. Cells were treated with BSA‐conjugated oleic acid (50 µm) to increase the intracellular lipid accumulation before sampling. B) Analysis of the proportion of different lipid metabolites. C) The level of diacylglycerol and monoacylglycerol in Vector and NPR1 overexpressing cells. Data presented as mean ± SD, *n* = 3, *p*‐values are calculated using multiple *t‐*tests. D) The level of triacylglycerol in Vector and NPR1 overexpressing cells. Data presented as mean ± SD, *n* = 3, *p*‐values are calculated using multiple t tests. E) Staining of lipid droplets in Vector and NPR1 overexpressing cells. Data presented as mean ± SD, *n* = 3, *p*‐values are calculated using unpaired Student's *t*‐tests. F) Staining of lipid droplets in Control and NPR1 knockdown cells. Data presented as mean ± SD, *n* = 3, *p*‐values are calculated using unpaired Student's *t*‐tests. G) The migration and invasion abilities of NPR1 overexpressing cells in a background of DMSO versus oligomycin or etomoxir exposure were analyzed. Scale bar = 50 µm. Data presented as mean ± SD, *n* = 3, *p*‐values are calculated using two‐way ANOVA tests. H) Schematic view of the popliteal lymph node metastasis mouse model treated by etomoxir. I) Representative images of the popliteal lymph node and primary tumor in the footpad. J) Images of popliteal lymph nodes. And statistical analysis of the volume and the presence of metastasis in popliteal lymph nodes, *n* = 7, Mann–Whitney test was used for comparing lymph node volumes, and Fisher's Exact test was used for comparing the presence of metastasis in lymph nodes. K) The presence of metastasis in the lymph node was confirmed by H&E and CK‐pan staining. Scale bar = 100 µm. ^*^ *p* < 0.05, ^**^ *p* < 0.01, ^***^ *p* < 0.001, ^****^ *p* <0.0001. Oligo: oligomycin. Eto: etomoxir.

Since proteomic data analysis reveals that NPR1 is involved in fatty acid metabolism, a pathway closely with tumor metastasis. we used targeted lipidomic to determine the effects of NPR1 overexpression on lipid metabolic remodeling in gastric cancer cells (**Figure** **4**A). We found that the different lipid metabolites (DLMs) were mainly glycerophospholipids (GP) and glycerolipids (GL) (Figure 4B). Specifically, the levels of triacylglycerol (TAG), diacylglycerol (DAG) and monoacylglycerol (MAG) were significantly lower in NPR1‐overexpressing cells compared to control cells (Figure 4C,D). Consistently, Lipid droplets, dynamic organelles that store lipids in the form of neutral lipids such as TAG, DAG, MAG and sterol lipids,^[^ ^18^ ^]^ were also reduced in the NPR1‐overexpressing cells compared to control cells (Figure 4E). Conversely, lipid droplet accumulation was observed in NPR1 knockdown cells (Figure 4F). Previous studies have showed that tumor cells consume lipid droplets for energy during migration and invasion.^[^ ^22^, ^33^ ^]^ Stored lipid droplets catabolized were also observed during GC cell migration and invasion (Figure S3, Supporting Information).

We hypothesized that fatty acids released from stored lipid droplets during NPR1‐induced lipolysis serve as a fuel source for invasion. To verify this, oligomycin, a potent inhibitor of mitochondrial ATP synthase, and etomoxir, a potent inhibitor of fatty acid beta‐oxidation that prevents mitochondrial import, were used to treat NPR1‐overexpressing gastric cancer cells. Both oligomycin and etomoxir significantly reduced the migration and invasion ability induced by NPR1 overexpression (Figure 4G).

We next explored the possibility of preventing metastasis by inhibiting FAO in gastric cancers with high NPR1 expression. NPR1‐high MGC803 cells were injected into the footpads of nude mice. 2 weeks post‐injection, mice were randomly assigned to two groups and received either vehicle (saline) or etomoxir (40 mg kg^−1^) for 4 weeks (Figure 4H). Subcutaneous administration of etomoxir effectively prevented LNM in mice injected with high NPR1‐expressing gastric cancer cells (Figure 4I–K). These results suggest that NPR1 promotes lipid droplet lipolysis, enhancing β‐oxidation and providing the energy necessary to fuel gastric cancer metastasis.

### 2.5 NPR1 Induces Lipid Droplet Lipolysis by Activating Lipase HSL

**Figure 5:**
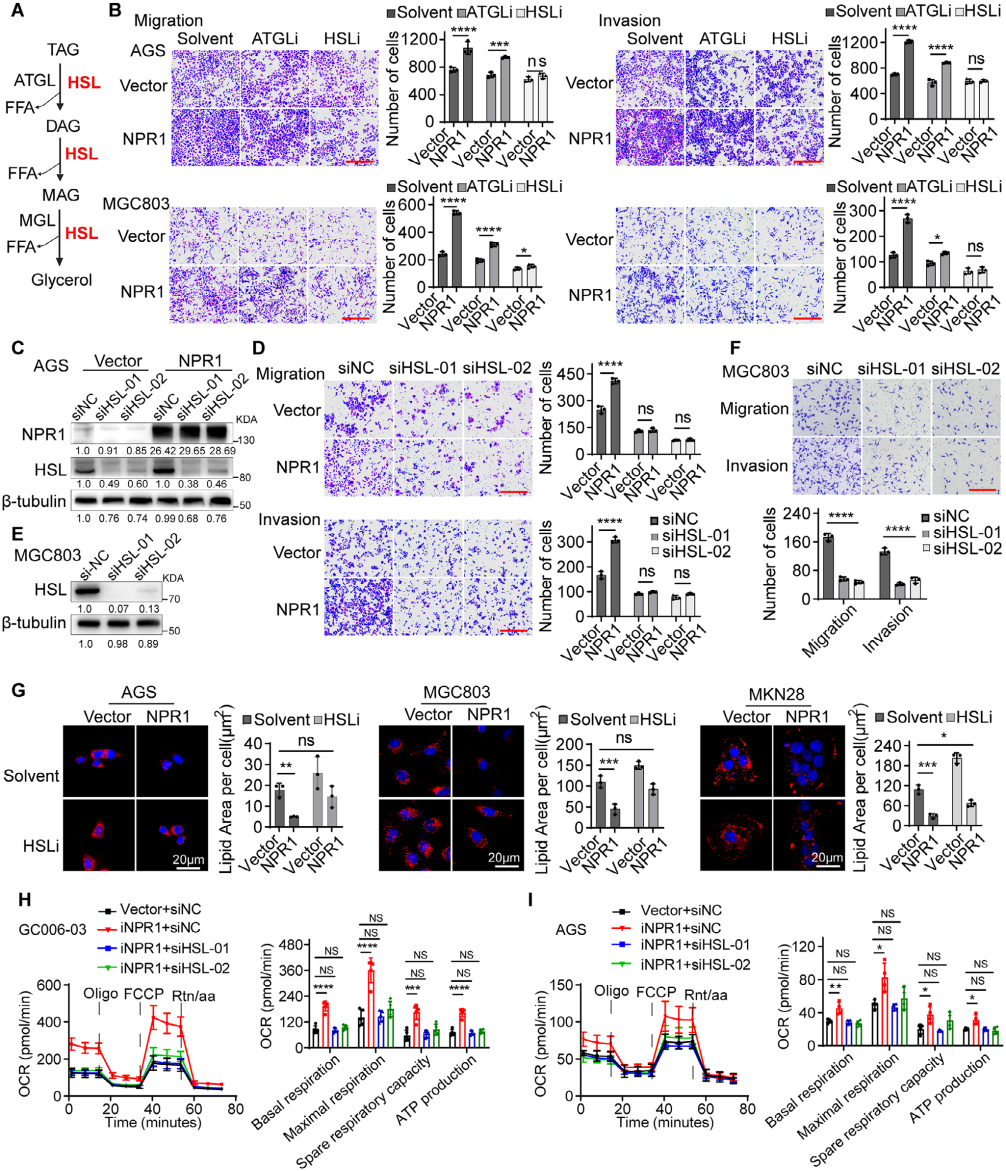
NPR1 promotes lipid droplet lipolysis by activating lipase HSL. A) Schematic view of the catabolism of lipid droplets. B) The migration and invasion abilities of NPR1 overexpressing cells in a background of DMSO versus ATGL inhibitor or HSL inhibitor exposure were analyzed. Scale bar = 50 µm. Data presented as mean ± SD, *n* = 3, *p*‐values are calculated using two‐way ANOVA. C) Western blot was used to evaluate the transfection efficiency of siHSL in Vector and NPR1 overexpression cells. D) The migration and invasion abilities of NPR1 overexpressing cells with HSL knockdown. Scale bar = 50 µm. Data presented as mean ± SD, *n* = 3, *p*‐values are calculated using two‐way ANOVA. E) Western blot was used to evaluate the transfection efficiency of siHSL in MGC803 cells. F) The migration and invasion abilities of MGC803 cells with HSL knockdown. Scale bar = 50 µm. Data presented as mean ± SD, *n* = 3, *p*‐values are calculated using two‐way ANOVA test. G) Staining of lipid droplets in Vector and NPR1 overexpressing cells in a background of HSL silence. Data presented as mean ± SD, *n* = 3, *p*‐values are calculated using unpaired Student's *t*‐test. H,I) OCR analysis of NPRI‐overexpressing GC006‐03 (H) and AGS (I) gastric cancer in the context of HSL knockdown, and statistical analysis of the mitochondrial respiration capacity and ATP production. Data presented as mean ± SD, *n* = 6 (H), *n* = 5 (I), *p*‐values are calculated using two‐way ANOVA test. ^*^ *p* < 0.05, ^**^ *p* < 0.01, ^***^ *p* < 0.001, ^****^ *p* < 0.0001. TAG: Triacylglycerol. DAG: diacylglycerol. MG: monoacylglycerol. ATGL: adipose triglyceride lipase. HSL: hormone‐sensitive lipase. MGL: monoglyceride lipase. FFA: Free fatty acid.

To explore which lipase is activated by NPR1 to regulate lipid droplet lipolysis, we focused on the three key lipases known to catalyze lipid droplet catabolism: adipose triglyceride lipase (ATGL), hormone‐sensitive lipase (HSL), and monoglyceride lipase (MGL).^[^ ^18^ ^]^ ATGL catalyzes the hydrolysis of TAG into DAG and free fatty acids. MGL converts monoglycerides into glycerol and free fatty acids, and HSL is capable of hydrolyzing TAG, DAG, and MAG (**Figure** **5**A).^[^ ^34^ ^]^ Our targeted lipidomic data revealed that levels of TAG, DAG, and MAG were all significantly reduced in NPR1‐overexpressing cells compared to control cells (Figure 4C,D), suggesting that ATGL and HSL may be critical for NPR1‐induced lipolysis.

To assess whether the lipolysis‐promoting effect of NPR1 depends on ATGL or HSL, we treated gastric cancer cells with Atglistatin (an ATGL inhibitor) or CAY 10499 (an HSL inhibitor). The HSL inhibitor markedly reversed NPR1‐induce migration and invasion, whereas the ATGL inhibitor had no significant effect (Figure 5B). Next, we knocked down HSL using siRNA in MGC803 cells, which express high levels of endogenous NPR1 (Figure 5C). Knockdown of HSL significantly inhibited cell migration and invasion in MGC803 cells (Figure 5D). Then we knockdown HSL in AGS cells stably expressing NPR1, and found that HSL silencing abrogated the enhanced invasion and migration of gastric cancer cell induced by NPR1 overexpression (Figure 5E,F). Moreover, HSL knockdown reversed the lipid droplet depletion caused by NPR1 overexpression (Figure 5G). Importantly, NPR1 overexpression significantly enhanced mitochondrial respiration capacity and ATP production, whereas HSL knockdown effectively rescued these NPR1‐induced mitochondrial metabolic enhancements, restoring OCR and ATP levels to baseline (Figure 5H,I). In addition, analysis of paired primary tumor and lymph node metastasis tissues revealed that HSL protein levels were higher in LVI tissues and lymph node metastasis tissues than in matched primary GC tissues (Figure S4, Supporting Information). These results indicated that NPR1 induces lipid droplet lipolysis by activating lipase HSL, thereby promoting gastric cancer metastasis.

### 2.6 PKG Directly Binds to HSL for NPR1‐Induced Activation of HSL

**Figure 6:**
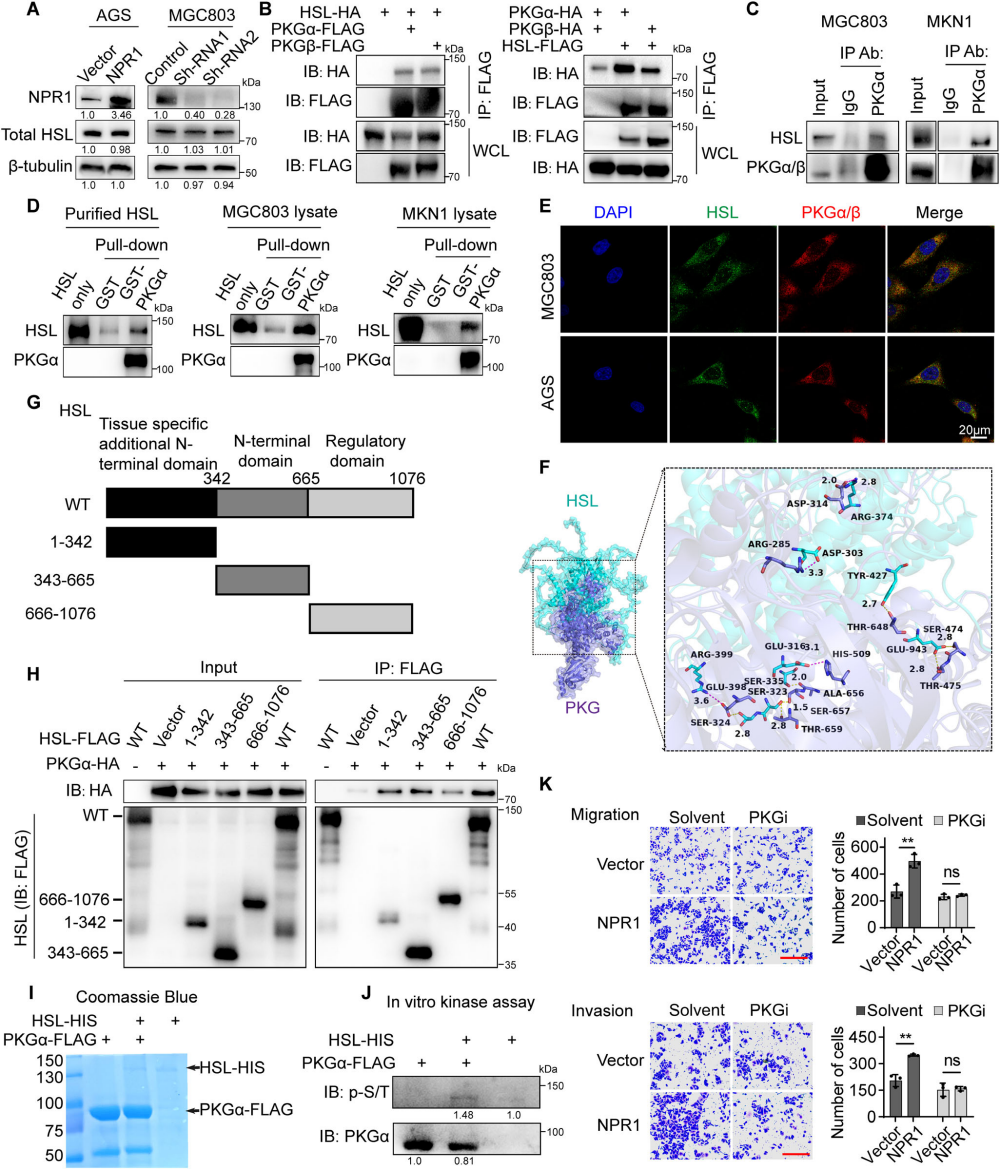
Protein kinase G (PKG) directly binds to HSL for NPR1‐induced activation of HSL. A) Western blotting was used to detect the protein level of HSL in NPR1 overexpressing or knockdown cells. B) Western blots on immunoprecipitation products using anti‐FLAG M2 affinity gel. HEK293T cells were transfected with HSL‐HA/PKG‐FLAG (left) or PKG‐HA/HSL‐FLAG (Right). 48 h later, cell lysates were prepared and used for the studies. C) Coimmunoprecipitation of endogenous PKGα with endogenous HSL from MGC803 and MKN1 cell lysates. D) GST pull‐down assays showing purified GST‐ PKGα binding with purified HSL and endogenous HSL in various cell lysates. E) Immunofluorescence staining showing colocalization of endogenous PKGα/β and HSL in MGC803 and AGS cells. F) Scheme of molecular docking. 3D structures of PKG (below) and HSL (above). G) Diagram for human HSL domain architecture. HSL contains a tissue specific additional N‐terminal domain (1 to 342), an N‐terminal domain (343 to 665), and a regulatory domain N‐terminal kinase domain (666 to 1076). H) Western blots on the input and immunoprecipitation products for interaction between PKGα and FLAG‐tagged truncated HSL. HEK293T cells were similarly transfected/processed, as described in (B). I) Coomassie blue staining of the purified PKGα‐FLAG protein from HEK293T cells and commercially available purified HSL‐HIS protein. J) Western blots on the products from in vitro kinase assays assessing affinity‐purified PKGα phosphorylation of HSL. PKGα proteins were overexpressed/purified using the EZview Red anti‐FLAG M2 affinity gel from the HEK293T cells transiently transfected with pRK5‐PKGα‐FLAG plasmid. K) The migration and invasion abilities of NPR1 overexpressing cells with PKG inhibition. Scale bar = 50 µm. Data presented as mean ± SD, *n* = 3, *p*‐values are calculated using two‐way ANOVA tests. ^*^ *p* < 0.05, ^**^ *p* < 0.01.

To determine how NPR1 regulates HSL, we detected HSL protein levels in NPR1‐overexpressing or knockdown cells. Western blot analysis showed that the protein levels of HSL were unaffected by NPR1 overexpression or knockdown (**Figure** **6**A), suggesting that NPR1 activates HSL through post‐translational modification rather than by altering its expression. Previous studies have shown that PKG, a primary downstream protein kinase of NPR1, is associated with HSL phosphorylation. However, there is no evidence showing HSL as a direct substrate of PKG.^[^ ^35^, ^36^ ^]^ Therefore, we sought to determine whether PKG is the key factor for NPR1‐induced HSL activation. The interaction between both PKG isoforms (PKGα and PKGβ) and HSL was detected by co‐immunoprecipitation (co‐IP) at both exogenous and endogenous levels in cells (Figure 6B,C), Additionally, pull‐down assays using GST‐tagged PKGα fusion protein demonstrated that GST‐PKGα, but not GST alone, directly binds to purified HSL protein. This interaction was further validated in MGC803 and MKN1 cell lysates, where GST‐PKGα effectively pulled down endogenous HSL (Figure 6D). Immunofluorescence assays confirmed the co‐localization between PKG and HSL in AGS cells and MGC803 cells (Figure 6E). These results indicate that PKG directly binds to HSL.

To investigate how PKG binds to HSL protein, we modeled the interaction between HSL (generated by Alphafold) and PKG (PDB 7T4T) by using structural docking. The analysis revealed multiple groups of residues involved in hydrogen bonding interactions between PKG and HSL (Figure 6F; Figure S5, Supporting Information). We next mapped the domain of HSL required for PKG (Figure 6G). Using the co‐IP assay, we found that the tissue specific additional N‐terminal domain of HSL (the 1 to 343 aa fragment of HSL, HSL [1‐343]) and the N‐terminal domain of HSL (the 344 to 665 aa fragment of HSL, HSL^344‐665^) exhibited high PKG binding affinity, while the regulatory domain (the 666 to 1076 aa fragment of HSL, HSL^666‐1076^) exhibited low PKG binding affinity (Figure 6H), which is consistent with the distribution of hydrogen bonding interactions obtained from the protein docking analysis.

In vitro kinase assays using purified PKG and HSL protein showed that PKG protein strongly phosphorylated HSL protein (Figure 6I,J). Furthermore, inhibition of PKG in NPR1‐overexpressing cells by KT 5832, a potent PKG inhibitor, significantly abrogated the enhanced invasion and migration of GC cells induced by NPR1 overexpression (Figure 6K). Collectively, these results strongly indicate that PKG directly binds to and phosphorylates HSL, modulating NPR1‐induced activation of HSL and promoting GC cell migration and invasion.

### 2.7 PKG Phosphorylates HSL at S855 and S951 for NPR1‐Induced Activation of HSL

**Figure 7:**
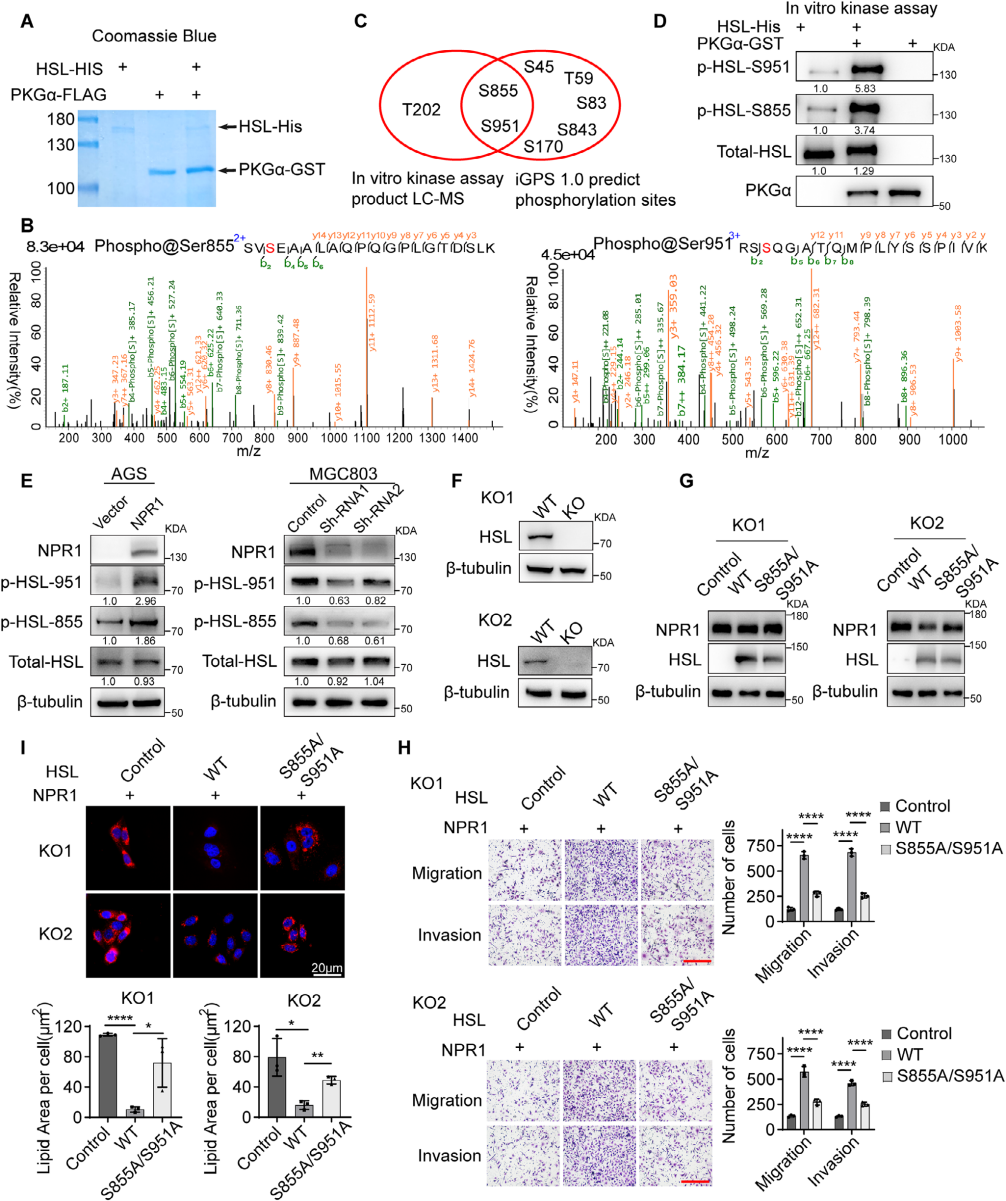
PKG phosphorylates HSL at S855 and S951 for NPR1‐induced activation of HSL. A) Coomassie blue staining of the commercially available purified PKGα‐GST protein and HSL‐HIS protein. B) Phosphorylation sites identified by LC‐MS/MS. C) Phosphorylation sites of PKG on HSL, Phosphorylation modification sites detected by LC‐MS using in vitro kinase reaction products. the reaction products (left), specific phosphorylation sites predicted by iGPS 1.0 software (right). D) Western blotting was used to confirm the phosphorylation of HSL by commercially available purified PKGα‐GST protein in vitro kinase assay product. E) Western blotting was used to confirm the phosphorylation level of HSL in NPR1 overexpressing or knockdown cells. F) Western blots on WT and HSL‐KO AGS cell lysates. G) Western blotting was used to detect the protein expression efficiency of NPR1, HSL^WT^, and HSL^S855A/S951A^ in two AGS HSL‐KO cells. H) The migration and invasion abilities of NPR1 overexpressing AGS HSL‐KO cells with HSL^WT^ or HSL^S855A/S951A^ overexpression. Scale bar = 50 µm. Data presented as mean ± SD, *n* = 3, *p*‐values are calculated using two‐way ANOVA tests. I) Staining of lipid droplets in NPR1 overexpressing AGS HSL‐KO cells with HSL^WT^ or HSL^S855A/S951A^ overexpression. Cells were loaded with oleic acid (50 µm) to increase the intracellular LD content before DNA transfection. Data presented as mean ± SD, *n* = 3, *p*‐values are calculated using two‐way ANOVA tests. ^*^ *p* < 0.05, ^**^ *p* < 0.01, ^***^ *p* < 0.001, ^****^ *p* < 0.0001.

To determine the PKG phosphorylation sites on HSL, we performed in vitro kinase assay using commercially available PKG protein overexpressed/purified from sf9 cells and commercially available purified HSL protein (**Figure** **7**A). Phosphorylation LC‐MS analysis of the reaction products showed that phosphorylation modifications at three HSL sites: threonine 202 (T202), serine 855 (S855) and serine 951 (S951) in the co‐incubation group compared to the control group (Figure 7B). Furthermore, we utilized the iGPS 1.0 software to predict the specific phosphorylation sites of PKG on HSL. We intersected these predicted phosphorylation sites with those identified from the LC‐MS analysis and obtained two overlapping phosphorylation sites, S855 and S951 (Figure 7C). In vitro kinase assay further confirmed that PKG strongly phosphorylated HSL at S855 and S951 (Figure 7D). Consistently, NPR1 overexpression in GC cells enhanced the phosphorylation levels of HSL at the S855 and S951 site. In contrast, decreased phosphorylation levels at these sites were observed in NPR1 knockdown cells (Figure 7E). These results suggest that PKG phosphorylates HSL at S855 and S951.

To determine whether the tumor‐promoting effect of NPR1 depends on the phosphorylation of HSL at S855 and S951 by PKG, we used the Lenti–Crispr/Cas9 system to knock out HSL in AGS cells. Western blot analysis confirmed efficient knockout of HSL in two monoclonal cell lines selected for further experiments (Figure 7F). Then, we introduced wild‐type HSL (HSL^WT^) or the S855A/S951A double mutant HSL (HSL^S855A/S951A^) into HSL‐KO cells (Figure 7G). Transwell assays showed that the introduction of wild‐type HSL, but not HSL^S855A/S951A^, rescued the impaired GC cell migration and invasion in HSL‐KO cells overexpressing NPR1 (Figure 7H). Nile Red staining revealed that the introduction of wild‐type HSL, but not HSL^S855A/S951A^, strongly depleted lipid droplets in HSL‐KO cells overexpressing NPR1 (Figure 7I). However, the introduction of HSL^S855A/S951A^ resulted in slightly increased cell migration, invasion and lipid droplets consumption compared to the control group cells, likely due to the activation of HSL by other kinases in the HSL‐KO cells (Figure 7H,I). Collectively, these results indicate that NPR1 promotes lipid droplet lipolysis, β‐oxidation, and GC metastasis through facilitating the phosphorylation of HSL at S855 and S951 by PKG.

### 2.8 Targeted Delivery of NPR1 siRNA by Engineered Exosome Mimics Effectively Suppressed the Gastric Cancer Metastasis

**Figure 8:**
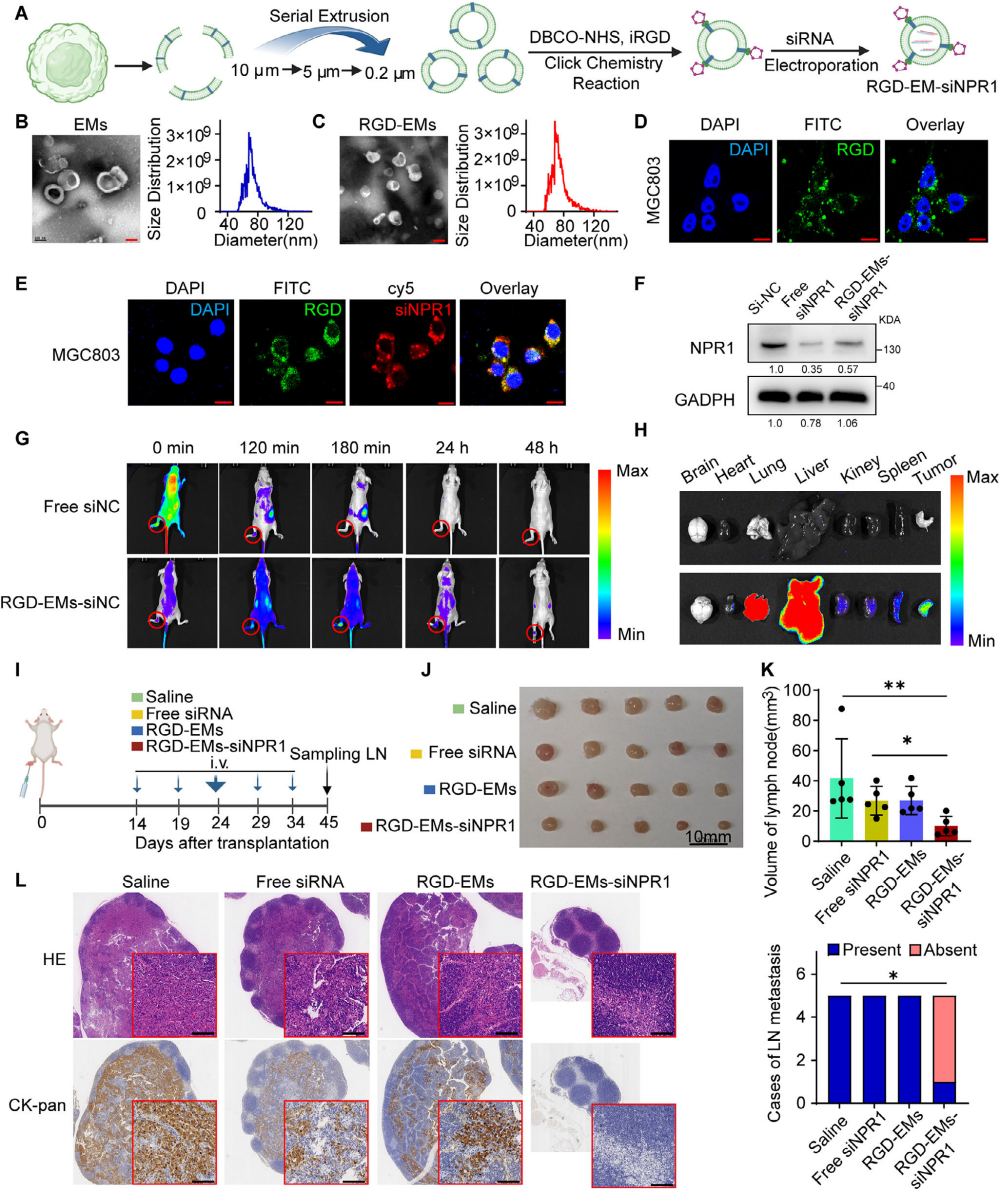
Targeted delivery of NPR1 siRNA by engineered exosome mimics effectively suppressed gastric cancer metastasis. A) Schematic view of the development of NPR1 siRNA (siNPR1) loaded engineered RGD‐exosome‐mimetic vesicles. B,C) EMs and RGD‐EMs Characterization, the morphology of EMs and RGD‐EMs was visualized by transmission electron microscopy (left), and the size distribution of EMs and RGD‐EMs was measured by a NanoFCM (right). Scale bar = 10 nm. D,E) Confocal Microscopy was used to examine the cellular uptake of RGD‐EMs or NPR1 siRNA‐loaded RGD‐EMs in MGC803 cells. Scale bar = 10 µm. F) Western blotting was used to examine the knockdown efficiency of expression efficiency of NPR1 in MGC803 cells with free NPR1 siRNA or NPR1 siRNA‐loaded RGD‐EMs treatment. G,H) In vivo distribution of bioengineered siRNA‐loaded RGD‐EMs in a popliteal lymph node metastasis mouse model. I) Schematic illustration of the treatment schedule in the popliteal lymph node metastasis mice model. J) Images of popliteal lymph nodes. K) Statistical analysis of the volume and the presence of metastasis in popliteal lymph nodes, *n* = 5, Mann–Whitney test was used for comparing lymph node volumes, and Fisher's Exact test was used for comparing the presence of metastasis in lymph nodes. L) The presence of metastasis in the lymph node was confirmed by H&E and CK‐pan staining. Scale bar = 100 µm. ^*^ *p* < 0.05, ^**^ *p* < 0.01.

Next, we attempted to investigate suitable strategies for inhibiting NPR1‐mediated tumor metastasis. Cell membrane‐derived exosome mimetics (EMs) are a drug delivery system for nucleic acids and other therapeutic molecules with several advantages, including good biocompatibility, low immunogenicity, high drug loading ability, excellent circulatory stability, and ease of production.^[^ ^37^, ^38^, ^39^, ^40^ ^]^ Arginine‐glycine‐aspartate (RGD) engineered exosomes have been proven to target tumors in vivo.^[^ ^41^, ^42^ ^]^ Here, we took advantage of the siRNA RGD‐engineered EMs delivery system to more effectively target NPR1 in vivo (**Figure** **8**A).

To prepare EMs, MGC803 cells were lysed, and the plasma membrane fraction was collected by ultracentrifugation. Then, the plasm membranes were sequentially extruded through 10, 5, and 0.2 µm polycarbonate membranes using a mini‐extruder, as previously described.^[^ ^38^ ^]^ These EMs were purified through ultracentrifugation. Their morphology was characterized by TEM, and size distribution was examined by nanoflow analysis (Figure 8B). Subsequently, RGD peptides were conjugated to the surface of EMs via a click chemistry reaction. These RGD‐EMs were further purified by ultracentrifugation, and their morphology was again characterized by TEM, with nanoflow analysis used to examine size distribution (Figure 8C). The morphologies of both EMs and RGD‐EMs resemble that of exosomes, with average diameters of ≈76 nm. siRNA was subsequently loaded into RGD‐EMs via electroporation.^[^ ^38^ ^]^

To investigate the uptake of RGD‐EMs by MGC803 cells, RGD was labeled with FITC, and siRNA was labeled with cy5. Confocal microscopy showed extensive internalization and accumulation of fluorescently labeled RGD‐EMs in the cytosol in MGC803 cells (Figure 8D). Similar results were observed in the RGD‐EMs‐NPR1 siRNA group (Figure 8E). Western blot showed that NPR1 siRNA delivered by RGD‐EMs reduced the protein levels of NPR1 in MGC803 cells (Figure 8F). Importantly, no significant cytotoxicity was detected in the RGD‐EMs treated cells (Figure S6A, Supporting Information). These results demonstrate the functionality of RGD‐EMs‐NPR1 siRNA in vitro.

To evaluate the tumor targeting effect and biodistribution of RGD‐EMs‐siRNA in vivo, BALB/C‐nu tumor‐bearing mice were injected with cy5 labeled free siNC or RGD‐EMs‐siNC via tail vein injection. At 180 min after i.v. injection, a strong accumulation of cy5 fluorescence was observed in the tumor area for the RGD‐EMs‐siNC group. In contrast, the free siNC group showed no fluorescence in the tumor area (Figure 8G). For in vivo biodistribution, mice's organs and tumors were harvested at 48 h after i.v. injection. The fluorescence intensity was accumulated in the tumor site in RGD‐EMs‐siNC group. In addition, RGD‐EMs‐siNC were widely distributed in the lung and liver, which may be attributed to the rich capillary networks within these organs (Figure 8H). These data suggest that RGD‐EMs can efficiently deliver siRNA to the tumor site in vivo.

To evaluate the anti‐tumor activity of RGD‐EMs‐NPR1 siRNA, we established a mouse popliteal lymph node metastasis model. The mice were randomly assigned to four groups, and received either saline, free NPR1 siRNA, RGD‐EMs or RGD‐EMs‐NPR1 siRNA every five days for four weeks (Figure 8I). Results showed that the free siRNA group and RGD‐EMs group had no significant effect on the volumes of popliteal LNs compared with the saline control group. On the other hand, the RGD‐EMs‐NPR1 siRNA group greatly reduced the volumes of popliteal LNs (Figure 8J,K). H&E and CK‐pan staining of popliteal LNs indicated that RGD‐EMs‐NPR1 siRNA treatment significantly inhibited the presence of LNM (Figure 8L). Moreover, H&E‐staining of organs showed no tissue damage following treatment (Extended Data Figure 5B). These results suggest that targeted delivery of NPR1 siRNA by engineered EMs effectively suppressed GC metastasis.

## 3 Discussion

Tumor metastasis is the leading cause of recurrence and mortality in gastric cancer patients. The high prevalence of LNM represents a major challenge in gastric cancer treatment, with a range from 10% to 42% in early gastric cancer.^[^ ^43^, ^44^ ^]^ However, the mechanisms underlying LNM in gastric cancer remain unclear. Recent studies underscore the pivotal roles of mitochondrial OXPHOS and FAO in driving tumor metastasis.^[^ ^6^, ^23^ ^]^ Our study showed that the protein level of NPR1 is significantly elevated in LVI and LNM compared to primary gastric tumors, and NPR1 expression is associated with poor prognosis. NPR1 promotes lipolysis of LD to release bioavailable FAs, which allow mitochondrial import of free FAs and upregulation of OXPHOS and ATP production, thus fueling gastric cancer cells’ metastasis. Mechanistically, NPR1 activates the downstream PKG, which directly binds to and phosphorylates HSL at the S855 and S951 sites, thereby promoting lipolysis and mitochondrial fatty acid oxidation. Furthermore, we developed engineered exosome‐mimetic vesicles for the targeted delivery of NPR1 siRNA, which effectively suppressed lymph node metastasis in gastric cancer. Our findings extend the understanding of lipid metabolism‐mediated tumor metastasis and may facilitate the development of novel strategies for preventing and treating lymph node metastasis in gastric cancer.

NPR1 is a transmembrane guanylate cyclase that has recently been shown to play an important role in tumor progression. Aberrant expression of NPR1 is associated with the malignant behaviors of several cancers, including gastric, esophageal, prostate cancer, and melanoma.^[^ ^27^, ^29^, ^45^, ^46^, ^47^ ^]^ However, its mechanism in cancer metastasis is unclear, and its function in gastric cancer metastasis and metabolism has not been investigated. Here, we identify NPR1 as a key driver of metabolic reprogramming in GC metastasis. NPR1 induced lipolysis of stored LDs to execute its key role in cancer, which is a new functional way for NPR1. Specifically, NPR1‐mediated activation of PKG directly phosphorylates and activates HSL at Ser855 and Ser951 to drive lipolysis, uncovering a new regulatory mechanism of metabolic reprogramming in GC metastasis. However, we did not detect a significant change in cell proliferation in NPR1‐overexpressing cells. Consistent with our findings, several studies have reported that upregulated FAO facilitates tumor metastasis without significantly affecting tumor proliferation.^[^ ^22^, ^48^, ^49^ ^]^ Studies have shown that lipid metabolism plays a key role in gastric cancer LNM.^[^ ^15^, ^16^ ^]^ We provided evidence that NPR1 promotes FAO to enhance gastric cancer cell invasive potential, and that the pro‐metastatic effect of NPR1 is not secondary to tumor proliferation enhancement. This discovery expands our understanding of NPR1's functional role in gastric cancer progression and highlights its potential as a therapeutic target for gastric cancer LNM.

Lipids are essential for maintaining the malignant phenotype of tumor cells at various stages of metastasis. Despite extensive research on lipid metabolism in cancer metastasis, most studies focus on lipid uptake rather than the mechanisms underlying the activation of stored lipid utilization. Our study identifies NPR1 as a critical regulator that initiates intracellular stored lipid oxidation metabolism in metastatic cancer cells. Consistent with our findings, several studies have shown that HSL activation enhances FAO and promotes cancer metastasis by facilitating lipid droplet degradation.^[^ ^22^, ^50^ ^]^ The NPR1‐HSL axis may represent a key pathway by which tumor cells upregulate OXPHOS during metastasis. Tumor cells acquire lipids from various sources. Primary tumor cells often increase lipid synthesis and storage.^[^ ^23^ ^]^ Other cell types in the tumor microenvironment, such as stromal cells, adipocytes, and fibroblasts, may also provide lipid to support tumor progression and metastasis.^[^ ^23^ ^]^ Metastatic tumor cells can acquire lipids from the microenvironment by upregulating lipid transport proteins.^[^ ^51^ ^]^ The lipid‐rich environment provides favorable conditions for the survival and colonization of metastatic tumor cells.^[^ ^51^, ^52^, ^53^ ^]^ Tumor cells take up excess lipids from the microenvironment and store them as lipid droplets. Lipid storage and utilization constitute a critical mechanism driving invasiveness in cancer cells. Excessive activation of HSL may deplete intracellular LD in tumor cells, thereby diminishing their metastatic potential. In contrast, during the metastatic cascade, LD utilization redirects cancer cell metabolism from glycolysis to mitochondrial OXPHOS, generating substantial ATP to fuel metastatic progression.^[^ ^22^ ^]^ During the metastatic cascade, HSL activation initiates LD lipolysis, amplifying OXPHOS‐driven metabolic output in metastatic cells and ultimately promoting metastasis.^[^ ^22^ ^]^ Thus, the role of HSL in cancer metastasis is multifaceted, potentially mediated through distinct mechanisms under varying contexts. The upregulation of lipid uptake and NPR1‐HSL‐mediated lipolysis‐based utilization of the stored lipids likely constitutes a critical mechanism driving gastric cancer cell metastasis.

Our data indicate that HSL activation‐induced lipid droplet breakdown plays a critical role in the metastatic cascade. HSL is activated via kinase‐mediated phosphorylation, which facilitates its translocation to LD surfaces, where it catalyzes the hydrolysis of glycerol esters, releasing free fatty acids (FAs).^[^ ^34^ ^]^ Its enzymatic activity predominantly correlates with phosphorylation status rather than mRNA abundance.^[^ ^34^, ^54^ ^]^ Our study reveals that the NPR1/PKG signaling axis activates HSL through post‐translational modifications to enhance LD lipolysis without altering total HSL protein expression. However, whether NPR1 regulates HSL expression/function through alternative pathways (e.g., transcriptional regulation or protein stabilization) remains unclear and warrants further investigation. PKA is a potent regulatory kinase in lipid metabolism. Studies have proved that PKA directly binds and phosphorylates HSL at Ser853, Ser950, and Ser951 sites.^[^ ^55^ ^]^ Given that PKA and PKG are closely related enzymes belonging to subgroup I of the AGC serine‐threonine protein kinase family, it has been hypothesized that PKG may also bind and phosphorylate HSL at these sites.^[^ ^34^ ^]^ However, direct evidence of protein interactions and in vitro kinase assay, and phosphorylation site identification has been lacking. In this study, we confirmed the direct binding of PKG to HSL through molecular experiments such as CO‐IP. Furthermore, in vitro kinase assays and LC‐MS analysis demonstrated that PKGα directly binds to and phosphorylates HSL at serine 855 and serine 951 sites. We proved that the site of PKG phosphorylation modifies HSL is different from that of PKA, which phosphorylates HSL at residues Ser853, Ser950, and Ser951. Since we observed that NPR1‐induced HSL activation was important to the metastatic potential of gastric cancer, these findings may contribute to our understanding of the regulatory mechanism underlying metabolic reprogramming in the context of metastasis.

EMs are a promising drug delivery system for nucleic acids and other therapeutic molecules with several advantages, including good biocompatibility, low immunogenicity, high drug loading ability, excellent circulatory stability, and ease of production.^[^ ^42^, ^56^ ^]^ Tumor cell‐derived extracellular vesicles exhibit preferential homing to parental tumor cells.^[^ ^57^, ^58^, ^59^ ^]^ This interesting property of tumor cell‐derived extracellular vesicles has also been found in metastatic cancers, which is known as organotropism.^[^ ^60^ ^]^ Meanwhile, tumor cell‐derived extracellular vesicles carry cell‐type‐specific proteins originating from their parent cells' plasma membranes (e.g., tetraspanins, integrins, CD11b and CD18 receptors) that confer an exceptional ability to interact with recipient cells.^[^ ^57^, ^58^, ^59^ ^]^ In this research, we conjugated the iRGD peptide to the surface of gastric cancer cell‐derived EMs through a click chemical reaction. iRGD, which acts as both an integrin‐homing peptide and a cell penetrating peptide, enhanced the drug delivery to the tumor microenvironment and enhanced tumor penetration.^[^ ^61^ ^]^ These RGD‐engineered EMs show exceptional gastric cancer cell uptake and targeting capability both in vitro and in vivo, suggesting the potential feasibility of this therapeutic strategy.

Taken together, our study suggests that NPR1 is an important player in gastric LNM.We reveal that the upregulation of NPR1 promotes lipolysis of LD to release bioavailable FAs, which allow mitochondrial import of free FAs and upregulation of OXPHOS, thereby fueling gastric cancer cell metastasis. This finding expands our understanding of NPR1's functional role in tumor metastasis and identifies NPR1 as a potential therapeutic target for treating gastric cancer metastasis. Of note, we proved that PKG directly binds to and phosphorylates HSL at S855 and S951, thereby mediating the NPR1‐induced lipolysis and OXPHOS. Targeted delivery of NPR1 siRNA by engineered EMs effectively suppressed the gastric cancer metastasis.

## 4 Experimental Section

### Sex as a Biological Variable

In this study, sex was not considered as a biological variable in the animal experiments. In human tumor tissue samples studies, sex was not considered as a biological variable.

### Cell Culture and Transfection

GES‐1, AGS and GC006‐03 cells were cultured in RPMI‐1640 medium (Gibco); HEK293T, MKN28, MGC803, MKN1 cells in DMEM medium (Gibco). Culture media were supplemented with 10% fetal bovine serum (Gibco) and 1% penicillin‐streptomycin (Gibco). All cells were cultured in a humidified incubator at 5% CO2 and 37 °C. The cell lines used in this study were validated by short tandem repeat profiling.

The siRNA duplex of HSL was purchased from RiboBio (Guangzhou, China). The siRNA duplex of NPR1 was designed and generated by Sangon (Shanghai, China). Transfection of siRNA was performed using Lipofectamine RNAiMAX reagent (ThermoFisher, United States). Transfection of DNA was performed using NEOFECT DNA transfection reagent (NEOFECT, China). NPR1 overexpression and NPR1 knockdown Lentivirus with resistance gene of purine were purchased from Genechem (Shanghai, China). Transient transfection and lentivirus packing/infection were done as previously reported.^[^ ^62^ ^]^ The siRNA, shRNA and sgRNA sequences are listed in Table S1 (Supporting Information).

### Plasmids

PiggyBac‐CMV‐NPR1, pCMV‐LIPE and pRK5 vector were purchased from MiaoLing Biology (Wuhan, China). pENTER‐CMV‐PRKG1v1, pENTER‐CMV‐PRKG1v2, pInducer20, and LentiCRISPR v2 vectors were purchased from Addgene (Watertown, United States). The pRK5‐HA, pRK5‐Flag, and pInducer20‐YF vectors were generated as previously described^[^ ^62^ ^]^ NPR1 cDNA was PCR amplified from PiggyBac‐CMV‐NPR1 and cloned into the pInducer20‐YF vector between the Mlu I and Xho I sites to produce the pInducer‐NPR1 vector. The CDS of PKGα, PKGβ, or HSL were PCR amplified from pENTER‐CMV‐PRKG1v1, pENTER‐CMV‐PRKG1v2, or pCMV‐LIPE and cloned into the pRK5‐HA or pRK5‐Flag vectors between the Mlu I and AsiSI sites to produce the pRK5‐PKGα‐HA, pRK5‐PKGα‐FLAG, pRK5‐PKGβ‐HA, pRK5‐PKGβ‐FLAG, pRK5‐HSL‐HA, and pRK5‐HSL‐FLAG vectors, respectively. The HSL CDS between Mlu I and AsiSI sites in the pRK5‐HSL‐FLAG vectors was replaced with the full‐length, truncated, and mutated CDS of HSL to produce pRK5‐HSL ^1‐343^‐FLAG, pRK5‐HSL^344‐665^‐FLAG, pRK5‐HSL^666‐1076^‐FLAG and pRK5‐HSL^S855A/S951A^‐FLAG vectors. The primers used for constructing plasmids are listed in Table S2 (Supporting Information).

### Generation of Knockout Cell Lines

The coding sequence of sgRNA was cloned into the lentiCRISPR‐v2 vector as previously described.^[^ ^62^ ^]^ The sgRNA‐expressing vectors were transfected into gastric cancer cells using NEOFECT DNA transfection reagent (NEOFECT, China). 48 h after transfection, cells were selected with 2 µg mL^−1^ puromycin (#M3637, Abmole, United States) for 1 day. After selection, single cells were sorted out by serial dilution in a 96‐well plate. Single clones were grown for 2 weeks and validated for loss of HSL via genomic sequencing and western blotting. The primers used for cloning sgRNA target sequences are listed in Table S2.

### Western Blot

Cells were lysed in RIPA buffer (#PC101, Epizyme, China) and quantified by BCA assay (#2J102, Epizyme, China). Equal amounts (5–20 µg) of protein were subjected to SDS‐PAGE gels and then transferred to PVDF for probing. Primary antibodies were listed in Table S3 (Supporting Information).

### Cell Migration and Invasion Assay

For transwell migration and invasion assays, cells (3 × 10^5^) were seeded in a 24‐well transwell chamber (8 µm pore size, Corning, United States). Cells suspended in serum‐free medium were seeded in the upper chambers with or without Matrigel coating (#356231, Corning, United States), and 600 µL of medium supplemented with 10% fetal bovine serum was added to the lower chamber. After incubation (24–48 h), cells on the bottom of the filter were fixed using 4% paraformaldehyde (PFA), stained using crystal violet, photographed, and counted under a microscope (Olympus Optical, Japan). For wound‐healing assays, cells were seeded into 6‐well plates and grown to confluency before a sterile pipette tip was used to scrape a straight line. Brightfield images were taken at the indicated times for each cell line. Each experiment was repeated three times. ImageJ software was used to determine the relative migration distance.

### 3D Tumor Spheroid Invasion Assay

The harvested cells (1 × 10^4^ cells) were suspended in growth factor‐reduced Matrigel (200 µL total volume, BD Biosciences, NY, USA), and then plated in wells. 500 µL of complete culture medium was gently added on top of the Matrigel. Cells were allowed to grow for 2 weeks and then imaged via microscopy (Olympus Optical, Japan).

### Immunofluorescence

Cells were fixed with 4% PFA for 20 min and washed with PBS three times. Then cells were permeabilized and blocked in Triton X‐100 in PBS (0.3%) containing 10% goat serum. Cells were incubated with appropriate primary antibodies at 4 °C overnight, followed by washing and incubation with secondary antibodies for 1 h at room temperature. Cell nuclei were stained with Hoechst 33342 at room temperature. Examined and photographed under a confocal microscope (LSM780, Zeiss, Germany).

### Neil Red Stain

The intracellular lipid droplets were stained with Nile Red (#HY‐D0718, MedChemExpress, USA). Cells were washed with PBS, then fixed with 4% PFA for 20 min and washed with PBS three times. Cells were incubated in the Nile Red working solution for 10 min at room temperature. After being washed with PBS three times, the Hoechst working solution was added to stain the nuclei. Finally, images were visualized using a fluorescence microscope (DMI8, Leica, Germany). Each experiment was repeated three times.

### Proteomics Analysis (Label‐Free)

Proteomics analysis was performed we previously reported.^[^ ^63^ ^]^ Briefly, cell samples were lysed with lysis buffer on ice for 30 min, followed by centrifugation (12 000 g, 20 min, and 4 °C). The protein solution was precipitated with acetone, and was reduced with 50 mm dithiothreitol for 1.5 h at 30°C. The protein solution was alkylated with 50 mm iodoacetamide for 15 min at room temperature in the dark. Then, 100 mm TEAB was added to urea in the protein sample that was then digested overnight by trypsin at 1:50 trypsin‐to‐protein mass ratio. Finally, the peptides were analyzed by LC‐MS/MS (Easy1200‐Faims Fusion Orbitrap, Thermo Fisher, USA).

### Metabolomics Analysis

The cell sample was placed in liquid nitrogen for 2 min, then thawed on ice for 5 min and vortexed. Repeat the first step 3 times, then centrifuge it at 12 000 rpm at 4 °C for 10 min. Homogenize it with 1 mL mixture (include methanol, MTBE, and internal standard mixture). Whirl the mixture for 15 min. Then add 200 µL of water and whirl the mixture for 1 min, and centrifuge it at 12,000 rpm at 4 °C for 10 min. Extract 500 µL supernatant and concentrate it. Dissolve powder with 200 µL reconstituted solution, then store in −80°C. Finally, take the dissolving solution into the sample bottle for LC‐MS/MS analysis. Lipid contents were detected by MetWare (http://www.metware.cn/) based on the AB Sciex QTRAP 6500 LC‐MS/MS platform.

### Oxygen Consumption Rate (OCR) Measurements

A Seahorse XF96 Flux Analyser (Seahorse Biosciences, USA) was used with Seahorse XF Cell Mito Stress Test kits and Seahorse XF96 FluxPak (Agilent Technologies, USA) reagents to measure the Oxygen Consumption Rate of GC cells. Cell suspensions were plated in XF96 microplates (2 × 10^4^/well) overnight, followed by baseline measurements. The cells were sequentially treated with oligomycin, FCCP to reversibly inhibit oxidative phosphorylation, and rotenone plus antimycin A (Rote/AA) to inhibit mitochondrial complexes I and III. Quantitation of the OCR was normalized by total protein level.

### Co‐Immunoprecipitation (Co‐IP)

Co‐IP was performed using EZview Red anti‐FLAG M2 affinity gel (Millipore Sigma, USA), as we previously reported.^[^ ^62^ ^]^ To detect endogenous PKG‐HSL interaction, MKN1 and MGC803 cells were treated with cGMP (100 µm) for 15 min before harvest. Cell lysates were prepared in lysis buffer (#P0013, Beyotime, China). Immunoprecipitation was performed using target antibody.

### GST Pull Down

The recombinant GST‐PKG protein was obtained from Sino Biological (#P78‐10BG, USA). And the recombinant HSL‐HIS protein was obtained from Cayman Chemical (#10664, USA). 500 ng GST or GST‐PKG protein were conjugated to GST‐purified magnetic beads (#C650031, Sangon Biotech, China) and incubated with 200 ng recombinant HSL or cell lysis in GST‐binding buffer containing cGMP (15 µm) at 4°C for 2 h, followed by washing 4 times for 8 min each with GST‐washing buffer (#C650031, Sangon Biotech, China) at 4 °C. The samples were then subjected to Western blotting.

### In Vitro Kinase Assay and Mass Spectrometry (MS)

In vitro kinase assay was performed as we previously reported.^[^ ^62^ ^]^ Briefly, the Recombinant HSL (#10664, Cayman Chemical, United States) was incubated with the recombinant PKG protein or control (as described above) in the in vitro kinase buffer containing cGMP (15 µm) at 30 °C for 1 h, and final incubation at 37 °C for 20 min. The reaction was stopped by adding 2 × SDS loading dye and boiling at 97°C for 5 min. We also used a similar protocol to examine the recombinant PKG protein (M2‐agarose bound, as described above) purified from cell lysis. The samples were then subjected to Western blotting for HSL phosphorylation at Ser^855^ (S855) and Ser^951^ (S951). For LC‐MS, the samples were run out on SDS gel, and examined by Coomassie blue staining. The gel containing HSL protein was cut and sent for LS‐MS analysis.

### Molecular Docking

The X‐ray crystal structures of PRKG1(7T4T) were retrieved from the Protein Data Bank. The predicted structures of LIPE were generated by Alphafold. To ensure the accuracy of the docking results, the protein was prepared by the AutoDockTools‐1.5.7,^[^ ^64^ ^]^ and the water molecules were manually eliminated from the protein, and the polar hydrogens were added. Docking Web Server (GRAMM) was used for protein‐protein docking.^[^ ^65^ ^]^ The resulting protein‐protein complex was also manually optimized by removing water and adding polar hydrogen by the AutoDockTools‐1.5.7. Finally, the protein‐protein interactions were predicted, and the protein‐protein interaction figure was generated by PyMOL (The PyMOL Molecular Graphics System, Version 3.0, Schrödinger, LLC.).

### Immunohistochemical Staining

Immunohistochemical staining (IHC) was performed as previously described.^[^ ^62^ ^]^ Briefly, slides were deparaffinized in xylene, rehydrated in descending concentrations of ethanol and PBS, and subjected to antigen retrieval by heated citrate buffer (pH 6.0). After antigen retrieval, slides were incubated with antibody at 4 °C overnight against NPR1 (1:50, Invitrogen, PA5‐29049), CK‐PAN (1:50, ZSGB‐BIO, ZM‐0069), respectively. Immunocomplexes were visualized by DAB method, and sections were counterstained with hematoxylin. IHC images were obtained on a microscope.

### Preparation and Purification of EMs

MGC803 cells were harvested with a cell scraper and washed with cold PBS twice. The cells were suspended in cold PBS with protease inhibitor cocktail. Freeze‐thaw and ultrasonic were used to disrupt cells. The entire solution was centrifuged at 3000 g for 15 min, collecting the supernatant. Next, the supernatant was centrifuged at 100 000 g for 30 min, collecting the pellet including plasma membranes. The pellet was suspended in cold PBS, followed by sequential extrusion through 10, 1, and 0.2 µm polycarbonate membrane filters (Whatman) 10 times using a liposome micro extruder (Avanti Polar Lipids). The obtained EMs were purified by gradient ultracentrifugation.

### Preparation and Purification of RGD‐EMs

RGD peptides were conjugated to the surface of EMs via a click chemistry reaction. Briefly, 10 µm DBCO‐NHS (#GC39241, Glpbio) was added to EMs solution (0.5 mg mL^−1^), and incubated away from light for 4 h at room temperature, all reactions were performed at pH 7.4. The reaction mixture was then centrifuged 3 times in a 100 kDa ultrafiltration tube (#910096, Merck Millipore) to remove excess DBCO‐NHS and obtain DBCO‐Dex/NV. Next, 1 µm c(RGDyK)‐N3 or FITC modified FITC‐c(RGDyK)‐N3 (synthesized by Scilight‐Peptide, Beijing, China), and 1 µm Cy5 azide (#GC12452, Glpbio) were added to the purified DBCO‐Dex/NV. The reaction was incubated away from light at 4 °C for 12 h in a rotating mixer. All reactions were performed at pH 7.4. The reaction mixture was then washed with cold PBS and centrifuged 3 times in a 100 kDa ultrafiltration tube. RGD‐EMs were resuspended with PBS and stored at −80°C for use.

### EMs Characterization

The size distribution and particle concentration of EMs and RGD‐EMs were measured by a NanoFCM (N30E, NanoFCM Inc., Xiamen, China) equipped with a green blue laser (488 nm) according to the manufacturer’ protocol. The size distribution and particle concentration data were analysed by the NanoFCM Software V1.17 (NanoFCM Inc., Xiamen, China). The morphology of EMs and RGD‐EMs was visualized by transmission electron microscopy (ai G2 Spirit, Thermo Fisher Scientific, Grand Island, USA) according to the manufacturer’ protocol.

### Preparation of siRNA‐Loaded RGD‐EMs

The siRNA was loaded into EMs and RGD‐EMs by using an electroporation system (Neon, Thermo Fisher, Grand Island, USA), as previously reported. Briefly, the RGD‐EMs obtained were mixed with the Neon electroporation buffer (#MPK10096, Thermo Fisher, Grand Island, USA) at a 1:1 ratio. siRNA was added to the mixture at a final concentration of siRNA: RGD‐EMs protein of 100 pmol µg^−1^ mL^−1^. Electroporation was then performed at 1300 V and 1 pulse. After electroporation, the mixture was incubated at 37 °C to allow EMs members to recover. After that, the reaction mixture was washed with cold PBS and centrifuged 3 times in a 100 kDa ultrafiltration tube to remove free siRNA outside the RGD‐EMs.

### Confocal Microscopy

After incubation with FITC‐RGD‐EMs and cy5‐siRNA loaded FITC‐RGD‐EMs for 4 h, cells were washed three times with cold PBS and fixed with 4% PFA for 20 min. Cell nuclei were stained with Hoechst 33 342 at room temperature. Examined and photographed under a confocal microscope (FV3000, Olympus, Japan).

### Cell Viability Assays

For cell viability assays, Cells were seeded in 96‐well plates (1000/well) and cultured at 37 °C in a humidified incubator. 10 µL of CCK8 solution (#GK10039, Glpbio) was added to the wells at the time points of 0, 24, 48, and 72 h, then the cells were incubated for 3 h at 37 °C. A microplate reader was used to measure the absorbance at 450 nm. Cell survival was reported as a percentage of the untreated control. For cell cytotoxicity assays, cells were seeded in 96‐well plates at a density of 3000 cells per well and treated with various concentrations of RGD‐EMs. Then, the 10 µL of CCK8 solution was added into the wells, and absorbance at 450 nm was measured by a microplate reader (MR9600, Benchmark, USA). Each experiment was repeated three times.

### In Vivo Studies

All animals were handled strictly following the Principles for the Utilization and Care of Vertebrate Animals and the Guide for the Care and Use of Laboratory Animals. ALB/c‐nu female mice were purchased from Shanghai Model Organisms (Shanghai, China). Mice aged 4–6 weeks and weighing 16–18 g were used.

### Biodistribution Study

The BALB/C‐nu mice with tumors in the footpads were used to examine the biodistribution of siRNA‐loaded RGD‐EMs. Cy5‐siRNA was loaded into RGD‐EMs as described above. Free cy5‐siRNA or RGD‐EMs loaded with Cy5‐siRNA were injected intravenously through the tail vein at a dosage of 15 mg kg^−1^. At the indicated time point after injection, the cy5 fluorescence in the tumor‐bearing mice was captured by the IVIS Spectrum Xenogen machine (Perkin Elmer, Waltham, USA). At 48 h after injection, mice were sacrificed through overanesthesia, and major organs, including brain, liver, spleen, lung, heart, kidney, and tumor, were collected. The fluorescence signals of cy5 were recorded.

### In Vivo Metastasis Models

To establish gastric cancer popliteal lymph node metastasis models, the indicated cell lines (5 × 10^5^/per mouse) were injected into the footpads of mice. After 7–8 weeks, the mice were euthanized, and the popliteal lymph nodes and primary tumors were collected. Popliteal lymph nodes were evaluated for metastatic lesions by H&E and IHC staining.

To establish gastric cancer hematogenous lung metastasis models, the indicated cell lines (1 × 10^6^/100 µL per mouse) were injected intravenously into the tail vein of mice. After 6 weeks, the mice were euthanized, and the lungs were collected. Lungs were evaluated for metastatic lesions by H&E staining.

To explore the potential application value of target gene therapy, the NPR1 siRNA‐loaded RGD‐EMs and negative control RGD‐EMs were produced as described above. The tumor‐bearing model was developed as described above. After 2 weeks, mice were randomly distributed into 4 groups. Animals were injected intravenously through the tail vein with NPR1 siRNA‐loaded RGD‐EMs, RGD‐EMs, free NPR1 siRNA, or PBS every 5 days. After 3 weeks, all mice were euthanized, and popliteal lymph nodes, primary tumors, and major organs (including liver and kidney) were collected for further detection. Popliteal lymph nodes were evaluated for metastatic lesions by H&E and IHC staining.

### Statistical Analysis

All statistical analyses were performed using GraphPad Prism 8.0.1 (GraphPad Software Inc., USA). Error bars represent standard deviations or standard error as indicated in the figure legends, and the n represents the number of samples or independent experiments as shown and described in the figure legends. Statistics are defined and detailed in the figure legends. Student's *t*‐tests or Chi‐Squared tests were used for pairwise comparisons, and the Wilcoxon signed‐rank test was used for non‐normally distributed data. One‐way or two‐way ANOVA was used for comparing multiple groups. Two‐tailed tests and an α of 0.05 were used for all statistical analyses. Survival analysis was carried out using univariate Cox and log‐rank tests.

### Study Approval

All animal experiments were approved by the Animal Experimentation Ethics Committee of the First Affiliated Hospital, Sun Yat‐sen University (approval numbers: [2023]005). All procedures involving the collection and application of human samples were approved by the Ethical Review Committee of the First Affiliated Hospital, Sun Yat‐sen University (approval numbers: [2024]567). All patients provided written informed consent and understood that their tissues would be used for research.

## Conflict of Interest

The authors declare no conflict of interest.

## Author Contributions

H.F., J.Z., H.C., and H.H. contributed equally to the work. H.F., J.Z., and H.C. participated in the manuscript preparation and formal analysis and performed most of the experiments. H.H. and R.X. contributed to cell culture and western blot. H.H., Y.C. and H.C. contributed to animal experiments. D.L. and J.Z supervised statistical analysis. Y.L., R.R. and J.O. provided intellectual feedback. D.Y., L.Z., and Q.C. reviewed and modified the manuscript. D.Y., L.Z., Q.C. and Y.H. conceived and directed the project.

## Supporting information



Supporting Information

## Data Availability

The data that support the findings of this study are openly available in Repository name at https://www.iprox.cn/page/home.html, reference number 10983000.
