# Supplementary material for: NPR1 Promotes Lipid Droplet Lipolysis to Enhance Mitochondrial Oxidative Phosphorylation and Fuel Gastric Cancer Metastasis
Source: Adv Sci (Weinh). 2025 Jun 20;12(37):e03233. doi: 10.1002/advs.202503233 (PMC12499470; doi:10.1002/advs.202503233)
Supplement: Supplementary file 1 — Supporting Information [file ADVS-12-e03233-s001.docx]

Supporting Information

**NPR1 Promotes Lipid Droplet** **Lipolysis to** **Enhance** **Mitochondrial Oxidative Phosphorylation and Fuel Gastric Cancer Metastasis**

*Huafeng Fu^1,6^, Jie Zhang^1^, Hengxing Chen^2^, Haobin Hou^1^, HuanJie Chen^5,6^, Rongman Xie^1^, Yanlei Chen^4,5^, Jian, Zhang^1^, Dehua Liu^1^, LePing Yan^2,4^, Rui Reis^7,8^, Joaquim Oliveira^7,8^, Yulong He^1,2,3^*, Li Zhong^2,4^*, Qinbo Cai^5,6^*, Dongjie Yang^1,2,3^**

Affiliation：

^1^Department of Gastrointestinal Surgery, Digestive Medicine Center, The Seventh Affiliated Hospital, Sun Yat-sen University, Shenzhen 518107, China

^2^Guangdong Provincial Key Laboratory of Digestive Cancer Research, Shenzhen, 518107, China

^3^Research Center for Diagnosis and Treatment of Gastric Cancer, Sun Yat sen University, Guangzhou, China

^4^Scientific Research Center, The Seventh Affiliated Hospital of Sun Yat-sen University, Shenzhen, 518107, PR China

^5^Center for Gastrointestinal Surgery, The First Affiliated Hospital, Sun Yat-sen University, Guangzhou 510080, China

^6^General Surgery laboratory, The First Affiliated Hospital, Sun Yat-sen University, Guangzhou, Guangdong, P. R. China

^7^3B’s Research Group, I3Bs – Research Institute on Biomaterials, Biodegradables and Biomimetics, University of Minho, Headquarters of the European Institute of Excellence on Tissue Engineering and Regenerative Medicine, AvePark, Zona Industrial da Gandra, 4805-017 Barco, Guimarães, Portugal;

^8^ ICVS/3B’s - PT Government Associate Laboratory, Braga/Guimarães, Portugal;

H.F., J.Z., H.C., and H.H. contributed equally to the work.

***Corresponding to:**

Prof. Yulong He, Electronic address: heyulong@mail.sysu.edu.cn.

Prof. Li Zhong, Electronic address: [zhongli@sysush.com](mailto:zhongli@sysush.com).

Prof. QinBo Cai. Electronic address: caiqinbo@mail2.sysu.edu.cn.

Prof. Dongjie Yang, Electronic address: [ydongj@mail.sysu.edu.cn](mailto:ydongj@mail.sysu.edu.cn).


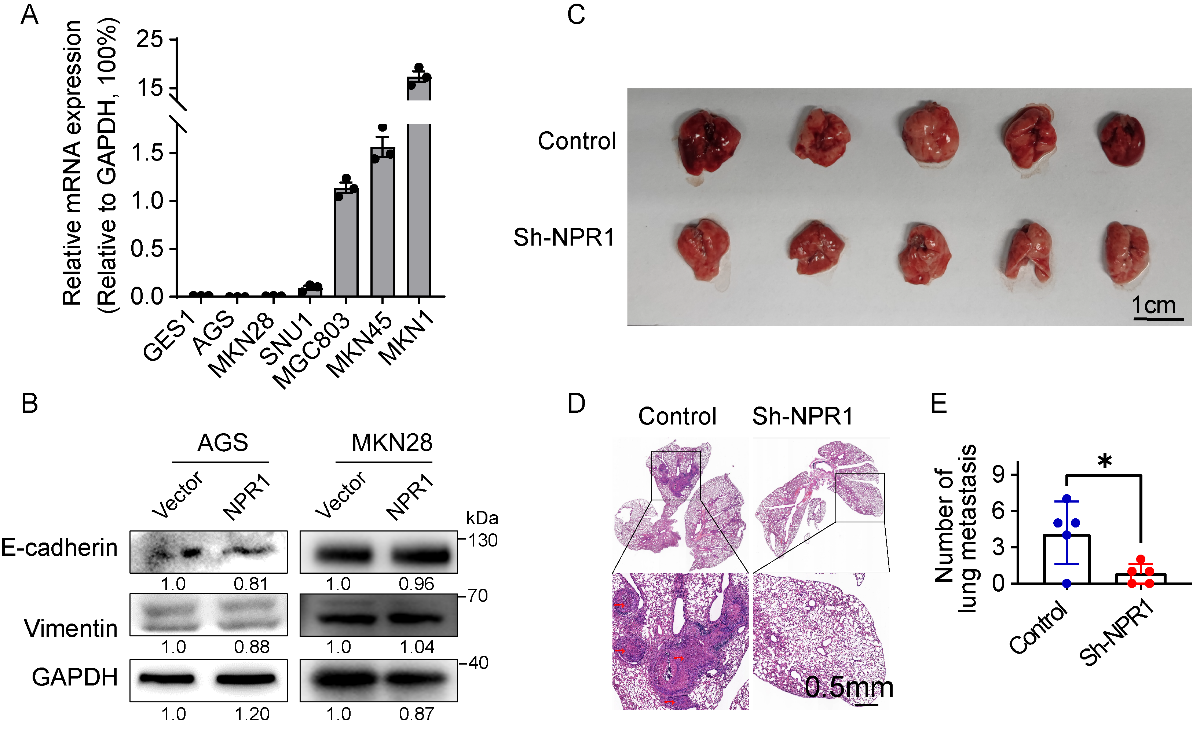


**Figure S1. NPR1 promotes gastric cancer cell metastasis. A.** The mRNA levels of NPR1 in several gastric cell lines; **B.** The protein levels of EMT markers in NPR1-overexpressing cells. **C.** The figure shows the gross view of the lung in mice; **D.** The figure shows a representative image of HE staining of lung sections; **E.** The figure shows the statistical analysis of the number of lung metastasis nodes between NPR1 knockdown and the control group, n = 5, statistical analysis was performed using the Mann-Whitney test. * *P* < 0.05.


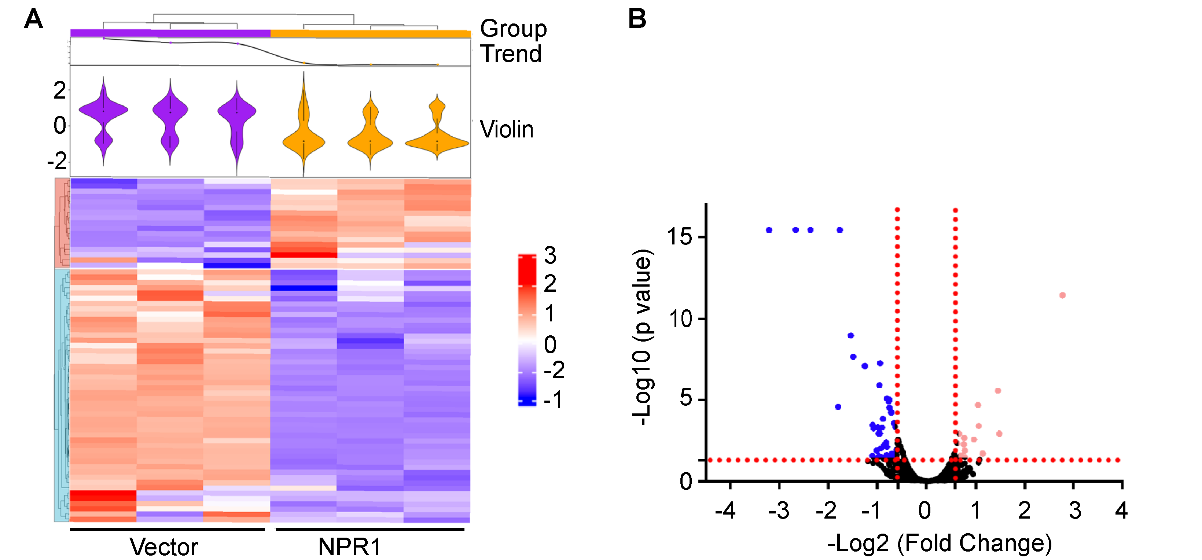


**Figure S2. Proteomic analysis on NPR1-overexpressing and control cells. A.** Cluster heat maps of protein profiling of NPR1 overexpression and control cells; **B.** Scatter plots of different proteins between the overexpression of NPR1 and the control cell groups, with 65 significantly different proteins identified.


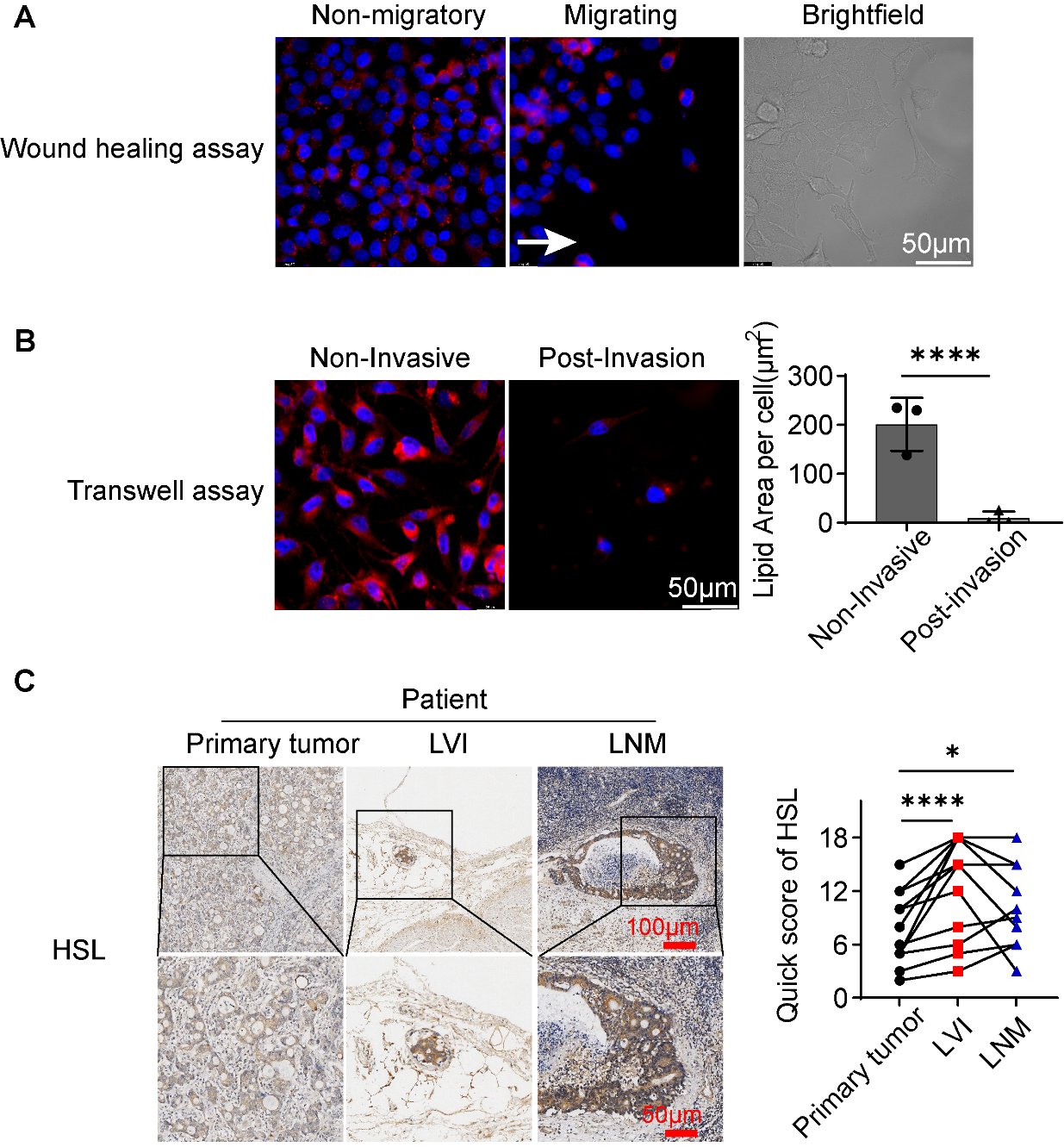


**Figure S3.** **Gastric cancer cells consume stored lipid droplet during migration and invasion.** **A.** In the wound healing assay, the intracellular lipid droplets of non-migratory cells and migrating cells are stained, and the arrow indicates the direction of cell migration. It can be observed that the highly motile leading-edge cells showed fewer lipid droplets than non-migratory tumor cells. **B.** In the Transwell assay, the intracellular lipid droplets of gastric cancer cells are stained. The number of lipid droplets in post-invasion cells was significantly lower than that in non-invasive tumor cells. Data presented as mean ± SD, n = 3, P-values are calculated using unpaired Student’s t tests.


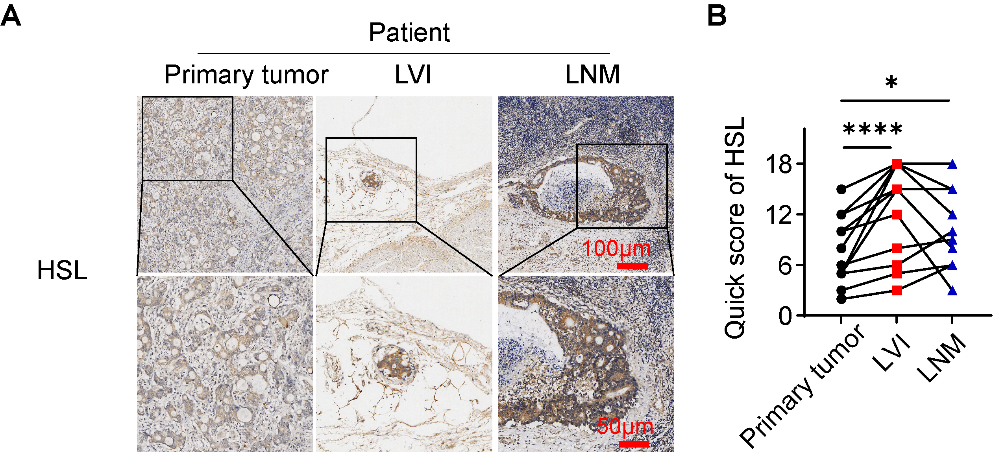


**Figure S4. HSL protein levels were higher in metastasis tissue. A.** Representative images of IHC staining for HSL expression in metastatic gastric cancer and primary tumors. **B.** Statistical analysis of the protein expression of HSL in metastatic gastric cancer and primary tumors. n = 16, P-values are calculated using Friedman's ANOVA tests. * *p* ＜0.05, ** *p* ＜0.01, *** *p* ＜0.001, **** *p* ＜0.0001.

LVI: lymphovascular invasion.

LNM: lymph node metastasis.


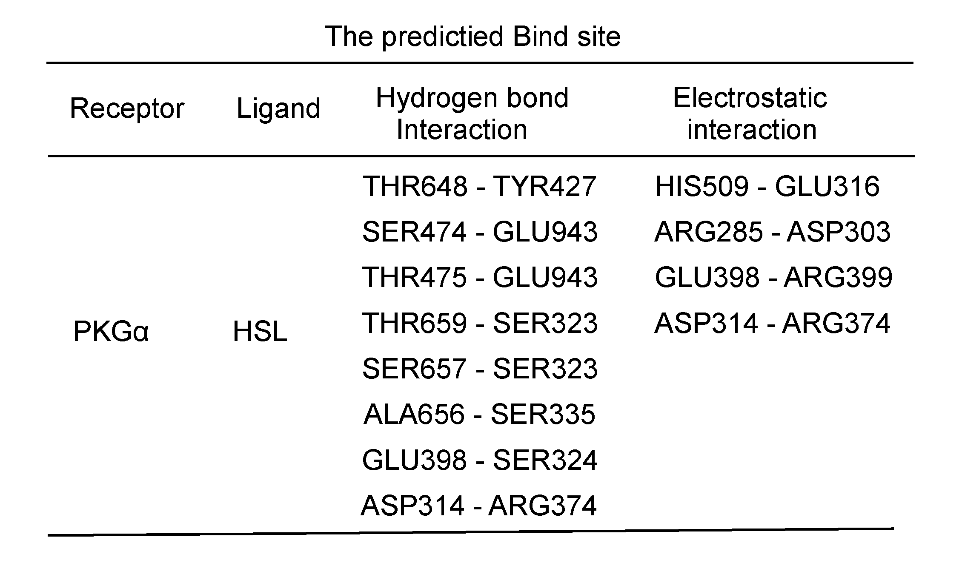


**Figure S5. The predicted bind site of PKGα and HSL.** By means of protein-protein interaction analysis in Pymol, all functional residues were identified and classified according to their interactions.


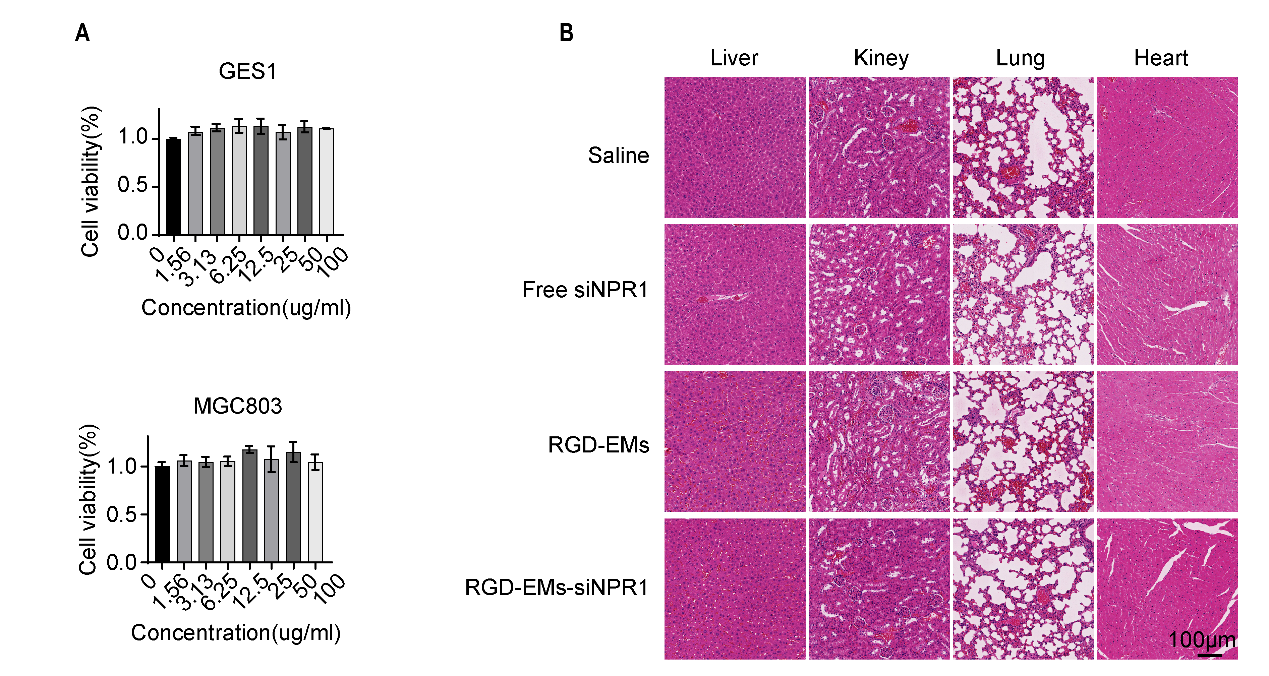


**Figure S6. Biosafety analysis of constructed exosomes mimetics. A.** No significant cytotoxicity was detected in the RGD-EMs treated cells. Data presented as mean ± SD, n = 5. **B.** H&E-staining of organs showed that no tissue damage following treatment.

**Supplementary Table1. The siRNA, shRNA and sgRNA sequences.**

| Gene | Sequence |
| --- | --- |
| sh-NPR1-1 | GCAAAGGCCGAGTTATCTACA |
| sh-NPR1-2 | GGGTTGTACTGAACTACAATG |
| siNPR1 | GCAAAGGCCGAGUUAUCUACA |
| siHSL-#1 | CCCTGATAGTGCACTTCCA |
| siHSL-#2 | CACAGACACTACCAGGATA |
| HSL-sgRNA1 | GCGTTTGTAGTGCTCCCCGA |
| HSL-sgRNA2 | GAAGAAGATGCTGCGGCGGT |

**Supplementary Table 2. Primers used in this study.**

| Clone | Primers |
| --- | --- |
| NPR1 into pInducer20-YF | F: AGTGCGATCGCCATGCCGGGGCCCCGGCGCCCC  R: CGACGCGTGCCTCGGGTGCTACTCCCCCT |
| PKGα into pRK5 | F: AGTGCGATCGCCATGAGCGAGCTAGAGGAAGAC  R: CGACGCGTGAAGTCTATATCCCATCCTGAGTTG |
| PKGβ into pRK5 | F: AGTGCGATCGCCATGGGCACCTTGCGGGATTTAC  R: CGACGCGTGAAGTCTATATCCCATCCTGAGTTG |
| HSL into pRK5 | F: AGTGCGATCGCCATGGAGCCAGGTTCTAAGTCAGTGTC  R: CGACGCGTGTGTCGCCCCCCGCAGCC |
| Truncates into pRK5 |  |
| HSL 1-342 aa | F: AGTGCGATCGCCATGGAGCCAGGTTCTAAGTCAGTGTC  R: CGACGCGTCCGTACACCGGCAAAAACGCC |
| HSL 343-662 aa | F: AGTGCGATCGCCATGGAGCAGGCGCTGGGGCTGGA  R: CGACGCGTGGATCTGGAGGTCTGGGCCAC |
| HSL 663-1076 aa | F: AGTGCGATCGCCATGCACGAGCCCTACCTCAAGAGCTG  R: CGACGCGTGTGTCGCCCCCCGCAGCC |
| Mutation |  |
| HSL S855A | F: GCCGCAGTGTGGCAGAAGCAGCACT  R: CGGCGTCACACCGTCTTCGTCGTGA |
| HSL S951A | F: CCACCCCCGACGCTCCGCACAGGGTGCCACACAGAT  R: CCACCCCCGACGCTCCGCACAGGGTGCCACACAGAT |
| Gene editing verification |  |
| HSL-sgRNA1 | F: TAGGCTGCGAGTAGAACCTG  R: CTGTCCCCATCCCTCTCTTG |
| HSL-sgRNA2 | F: TCCTGCACAAATCCCGCTAT  R: CACCAGGTGCCTTCATTGTG |

**Supplementary Table 3. Antibodies used in this study.**

| Species | Antibody | Dilution | Company |
| --- | --- | --- | --- |
| Human | NPR1 | WB 1:1000 | Invitrogen PA529049 |
| Human | HSL | WB 1:1000 | CST 4107 |
| Human | p-HSL(Ser855) | WB 1:1000 | CST 4137 |
| Human | p-HSL(Ser951) | WB 1:1000 | CST 45804 |
| Human | PKGα | WB 1:1000 | Santa cruz sc-393987 |
| Human | PKGα/β | WB 1:200 | Santa cruz sc-271766 |
| Human | β-tubulin | WB 1:2000 | CST 2128 |
| Human | GAPDH | WB 1:2000 | CST 5174 |
| Human | Anti-HA | WB 1:1000 | CST 3724 |
| Human | Anti-Flag | WB 1:1000 | CST 8146S |
| Human | Anti-p-S/T | WB 1:1000 | ECM PP2551 |

**Supplementary Table 4. Clinical characteristics and NPR1 protein expression.**

| Clinical characteristics | | Number(166) | | NPR1 protein expression | | *P* |
| --- | --- | --- | --- | --- | --- | --- |
|  |  |  |  | Low ( % ) | High ( % ) |  |
| Sex |  | |  |  |  | 0.5149 |
|  | Female | | 58 | 31(53.4) | 27(46.6) |  |
|  | Male | | 108 | 52(51.9) | 56(48.1) |  |
| Age(year) |  | |  |  |  | 0.6097 |
|  | ≤ 50 | | 49 | 23(46.9) | 26(53.1) |  |
|  | > 50 | | 117 | 60(51.3) | 57(48.7) |  |
| T stage |  | |  |  |  | **0.0016** |
|  | T 1 - 2 | | 32 | 24(75.0) | 8(25.0) |  |
|  | T 3 - 4 | | 134 | 59(44.0) | 75(56.0) |  |
| LNM |  | |  |  |  | **0.0079** |
|  | N 0 | | 43 | 29(67.4) | 14(32.6) |  |
|  | N 1-3 | | 123 | 54(43.9) | 69(56.1) |  |
| TNM stage |  | |  |  |  | **0.0010** |
|  | I | | 26 | 21(80.8) | 5(19.2) |  |
|  | II - III | | 140 | 62(44.3) | 78(55.7) |  |
| Differentiation |  | |  |  |  | 0.6270 |
|  | High | | 2 | 1(0.5) | 1(0.5) |  |
|  | Medium | | 62 | 34(54.8) | 28(45.2) |  |
|  | Low | | 102 | 48(47.1) | 54(52.9) |  |
| Max tumor diameter（cm） |  | |  |  |  | 0.0766 |
|  | ≤ 5 | | 105 | 58(55.2) | 47(44.8) |  |
|  | > 5 | | 61 | 25(41.0) | 36(59.0) |  |
